# Class I HDAC inhibitor entinostat synergizes with PLK1 inhibitors in *MYC*-amplified medulloblastoma cells

**DOI:** 10.1007/s11060-023-04319-1

**Published:** 2023-05-15

**Authors:** Gintvile Valinciute, Jonas Ecker, Florian Selt, Thomas Hielscher, Romain Sigaud, Johannes Ridinger, Venu Thatikonda, Charlotte Gatzweiler, Sarah Robinson, Julie Talbot, Flavia Bernardi, Daniel Picard, Mirjam Blattner-Johnson, Simone Schmid, David T. Jones, Cornelis M. van Tilburg, David Capper, Marcel Kool, Marc Remke, Ina Oehme, Stefan M. Pfister, Martine F. Roussel, Olivier Ayrault, Olaf Witt, Till Milde

**Affiliations:** 1 Hopp Children’s Cancer Center Heidelberg (KiTZ), Heidelberg, Germany; 2 Clinical Cooperation Unit Pediatric Oncology, German Cancer Research Center (DKFZ) and German Consortium for Translational Research (DKTK), Heidelberg, Germany; 3 KiTZ Clinical Trial Unit (ZIPO), Department of Pediatric Hematology and Oncology, Heidelberg University Hospital, Heidelberg, Germany; 4 Division of Biostatistics, German Cancer Research Center (DKFZ), Heidelberg, Germany; 5 Institut Curie, PSL Research University, CNRS UMR, INSERM, Orsay, France; 6 Université Paris Sud, Université Paris-Saclay, CNRS-UMR 3347 INSERM U1021, Orsay, France; 7 Department of Pediatric Oncology, Hematology and Clinical Immunology, Medical Faculty, University Hospital Düsseldorf, Düsseldorf, Germany; 8 Department of Pediatric Neuro-Oncogenomics, German Cancer Research Center (DKFZ), Heidelberg, Germany; 9 German Cancer Consortium (DKTK), partner site Essen/Düsseldorf, Düsseldorf, Germany; 10 Division of Pediatric Glioma Research, German Cancer Research Center (DKFZ), Heidelberg, Germany; 11 Department of Neuropathology, Charité - Universitätsmedizin Berlin, Berlin, Germany; 12 DKTK Partner Site, Berlin, Germany; 13 Division of Pediatric Neurooncology, German Cancer Research Center (DKFZ) and German Consortium for Translational Research (DKTK), Heidelberg, Germany; 14 Princess Máxima Center for Pediatric Oncology, Utrecht, The Netherlands; 15 Department of Tumor Cell Biology, St. Jude Children’s Research Hospital, Memphis, TN USA; 16 Present Address: Global Computational Biology and Digital Sciences, Boehringer Ingelheim RCV GmbH, Co KG, Doktor-Boehringer-Gasse 5-11, 1120 Vienna, Austria; 17 Hopp Children’s Cancer Center Heidelberg (KiTZ), CCU Pediatric Oncology B310, German Cancer Research Center (DKFZ), Im Neuenheimer Feld 280, 69120 Heidelberg, Germany

**Keywords:** Medulloblastoma, MYC, HDAC inhibitor, PLK1 inhibitor

## Abstract

**Purpose:**

We and others have demonstrated that *MYC*-amplified medulloblastoma (MB) cells are susceptible to class I histone deacetylase inhibitor (HDACi) treatment. However, single drug treatment with HDACi has shown limited clinical efficacy. We hypothesized that addition of a second compound acting synergistically with HDACi may enhance efficacy.

**Methods:**

We used a gene expression dataset to identify PLK1 as a second target in MB cells and validated the relevance of PLK1 in MB. We measured cell metabolic activity, viability, and cycle progression in MB cells after treatment with PLK1-specific inhibitors (PLK1i). Chou–Talalay synergy calculations were used to determine the nature of class I HDACi entinostat and PLK1i interaction which was validated. Finally, the clinical potential of the combination was assessed in the in vivo experiment.

**Results:**

*MYC*-amplified tumor cells are highly sensitive towards treatment with ATP-competitive PLK1i as a monotherapy. Entinostat and PLK1i in combination act synergistically in MYC-driven MB cells, exerting cytotoxic effects at clinically relevant concentrations. The downstream effect is exerted via MYC-related pathways, pointing out the potential of *MYC* amplification as a clinically feasible predictive biomarker for patient selection. While entinostat significantly extended survival of mice implanted with orthotopic *MYC*-amplified MB PDX, there was no evidence of the improvement of survival when treating the animals with the combination.

**Conclusion:**

The combination of entinostat and PLK1i showed synergistic interaction in vitro, but not in vivo. Therefore, further screening of blood–brain barrier penetrating PLK1i is warranted to determine the true potential of the combination as no on-target activity was observed after PLK1i volasertib treatment in vivo.

**Supplementary Information:**

The online version contains supplementary material available at 10.1007/s11060-023-04319-1.

## Introduction

Medulloblastoma (MB) is one of the most common pediatric malignant brain tumors [1], with substantially differing survival rates from nearly 100% to below 50% 5-year overall survival (OS) [2] depending on molecular group (WNT, SHH, group 3 and group 4) [3]. Each of the groups can be differentiated further [2, 4, 5]; in particular group 3/4 MBs with eight subgroups (I–VIII) [5, 6]. Advanced therapeutic strategies are needed for the more aggressive subgroups of MB, e.g. group 3/4 subgroup II with *MYC*-amplification.

*MYC* amplification is one of the most critical determinants for poor progress-free (PFS) and OS [7]. MYC is a transcription factor (TF) frequently driving various malignancies [8]. As with other TFs, direct targeting of MYC has been a challenge [8]. Therefore, targeting of the MYC transcriptional complex or the transcription of the *MYC* gene remain important strategies against MYC-driven tumors [8].

Histone deacetylases (HDACs) are enzymes that remove the acetyl group of lysine in histones and other proteins. The four classes of HDACs (I-IV) differ in their structure, localization, and targets [9]. High sensitivity of class I HDAC inhibitor (HDACi) treatment in *MYC*-amplified MB has been described [10, 11]. HDACis directly interfere with the MYC transcriptional program in MYC-driven MB [12]. While HDACis showed only moderate clinical success as single therapeutics [13], combination therapy shows preclinical activity [11].

Polo-like kinases (PLKs) [14] are serine/threonine phosphorylation-catalyzing enzymes. There are five PLKs (PLK1-5), with PLK1 being the most extensively studied family member due to its distinct role in cell cycle regulation. PLK1 is upregulated in various tumor entities, indicating oncogenic activity, but evidence of PLK1 functioning as a tumor suppressor has been published as well [15]. PLK1 inhibitors (PLK1i) have been used in pre-clinical [16, 17] and clinical [18] studies for over a decade, yet so far no PLK1i has been approved for clinical use to date, mainly due to less-than-expected clinical efficacy and dose-limiting toxicity. Applying PLK1i in synergistic combination treatments of sensitive cancer entities could lead to both increased efficacy and decreased toxicity.

Here, we identify PLK1 as a potential co-target for a combination therapy with the class I HDACi entinostat in *MYC*-amplified medulloblastoma and investigate synergistic interactions in vitro and in vivo.

## Materials and methods

### Cell culture

All cell lines were cultured as previously described. MB: MED8A, UW228-2 and ONS-76 [19], HD-MB03 [20] and D458 [21]. The *MYC* amplification status was confirmed by methylation-array-based copy number plots [22] (Suppl. Figure 1), which were performed as previously described [23]. Non-transformed human foreskin fibroblasts VH7 [24]. All cell lines were monitored for contamination and authenticated by Multiplex cell Contamination Test (McCT) service (Heidelberg, Germany) as described [25]. Short-term patient-derived xenograft (PDX) cell culture is described in Supplementary materials and methods.

### Drugs

Inhibitors used in vitro and in vivo are summarized in the Supplementary Table 1 and described in Supplementary materials and methods.

### siRNA-mediated PLK1 knock-down

Transfection with four PLK1-targeting siRNAs (Cat. No. 1027416, Hs_PLK1_4, 6, 7, 11 FlexiTube siRNA, Qiagen, Hilden, Germany) was conducted according to the manufacturer’s instructions using HiPerfect transfection reagent (Qiagen), each mix containing two of the PLK1 targeting siRNAs (mix 1: Hs_PLK1_4 and 6; mix 2: Hs_PLK1_7 and 11). Allstars (Qiagen) and RISC-free (Horizon Discovery, Waterbeach, UK) were used as controls.

### Animal studies

All animal experiments were conducted in accordance with ethical and legal regulations for animal welfare and approved by Regierungspräsidium Karlsruhe (G-270/19; Karlsruhe, Germany) and Animal Care and Use Committee at St. Jude Children’s Research Hospital. For details on in vivo studies see Supplementary materials and methods.

### WST-8 metabolic activity assay and viability analysis

5000 cells/well were seeded on a 96-well plate (Corning Inc., Corning, NY, USA) 24 h before treatment. Drugs were administered using the Tecan 300e Digital Dispenser (Tecan Group Ltd, Mannedorf, Switzerland). After 72 h incubation, WST-8 tetrazolium salt-based assay (Roche, Basel, Swizterland) was performed following manufacturer’s instructions. Cell number and viability was determined after 72 h treatment using trypan blue exclusion method in an automated Vicell XR cell counter (Beckman Coulter, Brea, CA, USA). In order to differentiate both assays within this manuscript, “cell viability” refers to the trypan blue exclusion method and “metabolic activity” refers to the WST-8 assay.

### Cell cycle analysis by flow cytometry

Cells were fixed in ice-cold 70% ethanol for 1 h, washed with 38 mM Na-citrate buffer and stained with 50 µg/mL propidium iodide (PI) containing 50 µg/mL RNAse A (Sigma-Aldrich) at 37 °C for 20 min. Measurement of DNA content was conducted using BD FACSCanto II platform (BD Bioscience, Franklin Lakes, NJ, USA), the data was analyzed using FlowJo™ v10.6.1 software (BD Bioscience).

### Caspase-3-like activity assay

Caspase-3-like activity was measured using the caspase-3 fluorometric assay kit (Biovision Inc., Milpitas, CA, USA) as described previously [19] after 24 or 48 h of treatment. For a positive control, cells were exposed to UV irradiation (35 mJ/cm^2^) 16 h before collection.

### RNA isolation, cDNA synthesis and quantitative reverse transcription real-time PCR (qRT-PCR)

RNA isolation, cDNA synthesis and qRT-PCR were performed and analyzed as published previously [19], using primers indicated in Supplementary Table 2 (Qiagen). Normal cerebellum RNA was used as control (Clontech, Moutain View, CA, USA).

### Immunoblotting

Immunoblotting was conducted as published previously [12]. Antibodies are listed in Supplementary Table 3. Images were acquired and enhanced with Azure c400 imaging system (Azure Biosystems Inc., Dublin, CA, USA) and cropped using Inkscape 0.92.4 software (open source). Protein expression was quantified using InfanView v4.54 (Irfan Skilijan) and ImageJ v1.52 (NIH, Bethesda, MD, USA). Protein expression was background-adjusted and normalized.

### Datasets used for gene expression, ChIP and protein abundance analysis

For details on gene expression, ChIP and protein data sets, see Supplementary materials and methods.

### Gene expression profiling and gene set enrichment analysis

Total RNA was isolated using RNeasy Mini Kit (Qiagen) after 6 h of 5 µM entinostat, 1 µM volasertib, or combinatorial treatment. Microarray analysis was done at the Genomics and Proteomics Core Facility at the German Cancer Research Center (DKFZ) using the Affymetrix Human U133 Plus 2.0 GeneChip according to the manufacturer’s instructions. Data was analysed as described in the Supplementary materials and methods.

### Data Availability

Gene expression profiling data is available in GEO database (GSE220748).

### Statistical analysis and data visualization

All experiments were performed in at least three biological replicates. All data is depicted in mean ± SD, unless otherwise indicated. For details on statistical evaluation and data visualization please see Supplementary materials and methods.

## Results

### The MYC target gene *PLK1* is downregulated upon class I HDAC inhibition

**Fig. 1:**
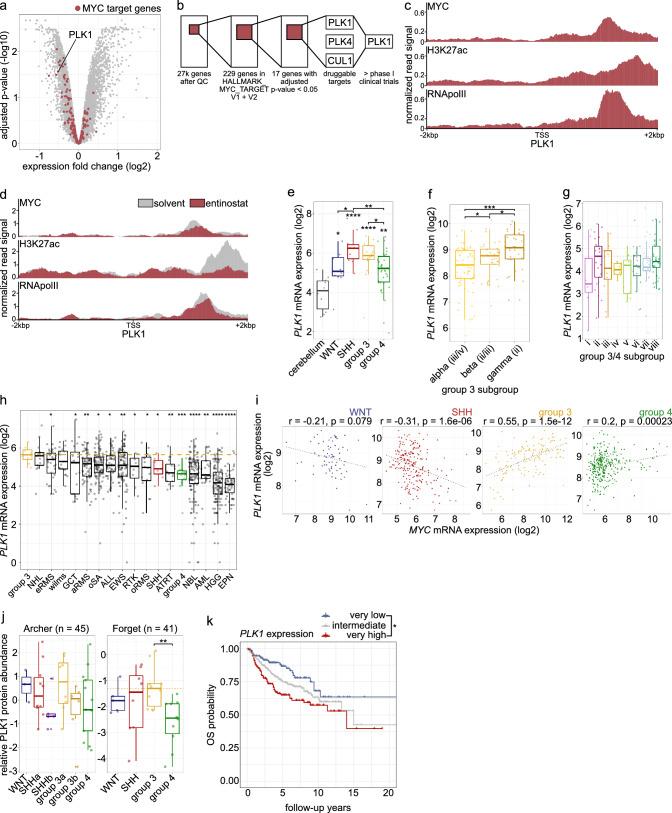
PLK1 is a relevant target in MB. **a** Volcano plot showing differential mRNA expression in HD-MB03 MB cells treated with 5 µM entinostat for 6 h, MYC target genes (HALLMARK_MYC_TARGET_V1 and V2 [26]) are marked in dark red. **b** Target gene filtering scheme. **c** MYC, H3K27ac and RNAPolII ChIP signal peaks around PLK1 TSS in primary MB tumors [12]. **d** MYC, H3K27ac and RNAPolII ChIP signal peaks around PLK1 TSS in HD-MB03 MB cells treated with solvent or 5 µM entinostat [12]. **e** *PLK1* mRNA expression in groups of MB (MB dataset: Gilbertson (n = 76) [48], cerebellum dataset: Kool (n = 10)). **f** *PLK1* mRNA expression in three group 3 subgroups of MB (Cavalli (n = 763) [2], equivalent subgroup in parentheses as suggested previously [6]). **g** *PLK1* mRNA expression in MB group 3 and 4 subgroups of medulloblastoma (Pfister (n = 223) [5], difference from the reference subgroup II is not significant). **h** PLK1 mRNA expression in pediatric cancer entities in INFORM database [27] (yellow line: group 3 MB median value). **i ***PLK1* and *MYC* mRNA expression correlation analysis in WNT, SHH, group 3 and group 4 of MB (Cavalli (n = 763) [2]; linear regression analysis with Pearson correlation coefficient). **j** Relative PLK1 protein abundance in Archer [28] and Forget [29] datasets (difference to the reference group 3a is not significant). **k** Overall survival (OS) differences between *PLK1* mRNA high (above Q3), low (below Q1) and intermediate expressing tumor patients (Cavalli (n = 763) [2]; Log Rank test). *ALL* acute lymphoblastic leukemia. *AML* acute myeloid leukemia. *ATRT* atypical teratoid rhabdoid tumor. *EPN* ependymoma. *GCT* germ cell tumors. *HGG* high grade glioma. *NBL* neuroblastoma. *NHL* non-Hodgkin lymphoma. *RTK* rhabdoid tumor of kidney. *EWS* Ewing’s sarcoma. *a/e/oRMS* alveolic/embryonal/other rhabdomyosarcoma. *oSA* other sarcoma. *SHH* sonic hedgehog MB. *p < 0.05; **p < 0.01; ***p < 0.001; ****p < 0.00001; ns or no indication: not significant

To identify MYC-driven genes downregulated upon class I HDAC inhibition, representing potential drug targets in combination with HDACis, we analyzed the regulation of the MYC target gene sets HALLMARK_MYC_TARGET_V1 and V2 [26] (229 genes) upon treatment of the *MYC*-amplified MB cell line HD-MB03 with the class I HDAC inhibitor entinostat (Fig. 1a). 17/229 (7.4%) MYC target genes were significantly regulated by entinostat treatment (Suppl. Table 4). Filtering for genes coding for proteins targetable with small molecule inhibitors identified three genes: PLK1, PLK4 and CUL1 (Fig. 1b). Of these three genes, only PLK1 and PLK4 inhibitors were in clinical trials at the time. Therefore, we chose to investigate PLK1 further because PLK1is were clinically the most advanced compounds (phase 3). PLK1 mRNA (Suppl. Fig. 2a) and protein (Suppl. Fig. 2b) expression were reduced upon entinostat treatment (at the same concentration as was used in the screen) (Fig. 1a). Enrichment of MYC, H3K27ac, and RNAPolII on the PLK1 promoter in three primary *MYC*-amplified group 3 MB samples (summary: Fig. 1c; individual tumors: Suppl. Fig. 2c–e) confirmed PLK1 to be a MYC activated gene (MAG) in human MB. Treatment of the *MYC*-amplified MB cell line HD-MB03 with entinostat led to a reduction of MYC, H3K27ac, and RNA PolII binding (Fig. 1d), thus confirming (as expected for MAGs) the transcriptional regulation of the MYC-target gene PLK1 upon class I HDAC inhibition. Thus, PLK1 is a MYC target gene regulated upon class I HDAC inhibition and could be a suitable target for combination with HDACis.

### The target PLK1 is expressed in *MYC*-amplified cell line models and primary tumors

To investigate the PLK1 presence, we analyzed its mRNA and protein expression in both primary samples and cell models of MB.

The analysis of *PLK1* mRNA expression in publicly available MB gene expression data sets showed significantly higher expression in MBs compared to cerebellum in all 4 molecular groups, particularly in SHH and group 3 (Gilbertson, n = 76, Fig. 1e). *PLK1* mRNA expression levels were highest in group 3 subgroup ii (equivalent to gamma, Cavalli, n = 763, Fig. 1f, Pfister, n = 223, Fig. 1g) associated with *MYC*-amplification [2, 5]. Recurrent group 3 MB showed the highest *PLK1* mRNA expression compared to other recurrent pediatric tumors analyzed in the INFORM database [27] (Fig. 1h). A significant positive correlation of *PLK1* to *MYC* mRNA expression with an r-value > 0.5 was observed in group 3 MB only (Fig. 1i). Relative PLK1 protein abundance was the highest in the subgroups associated with *MYC* amplification, G3a [28], and group 3 [29] (Fig. 1j). Finally, very high (above Q3) *PLK1* mRNA expression in pan-MB cohort was significantly associated with lower OS (Fig. 1k), further emphasizing the role of PLK1 as an oncogene in MB. In group 3 (Fig. 1k, Suppl. Fig. 3a) as well as group 3γ (Suppl. Fig. 3b), *PLK1* mRNA expression stratified patients with better (very low *PLK1* expression) or poorer survival (very high *PLK1* expression), while no such association was seen for *PLK2* and *4* (Suppl. Fig. 3c, d).

Analysis of the *MYC*-amplified and non-amplified (Suppl. Fig. 3f) cell line models used in this study revealed high *PLK1* mRNA expression compared to normal tissue (Suppl. Fig. 3e). Investigation of PLK1 protein in MB cells showed similar PLK1 expression in all tested cell lines (Suppl. Fig. 3g). No MYC status-dependent differences in doubling time were noted in all models investigated (Suppl. Figure 4).

In summary, we conclude that PLK1 is a valid target in *MYC*-driven background, and that the models are suitable for studying PLK1 targeting.

### *MYC*-amplified MB cell lines are more sensitive to PLK1 inhibition than *MYC* non-amplified MB cell lines

**Fig. 2:**
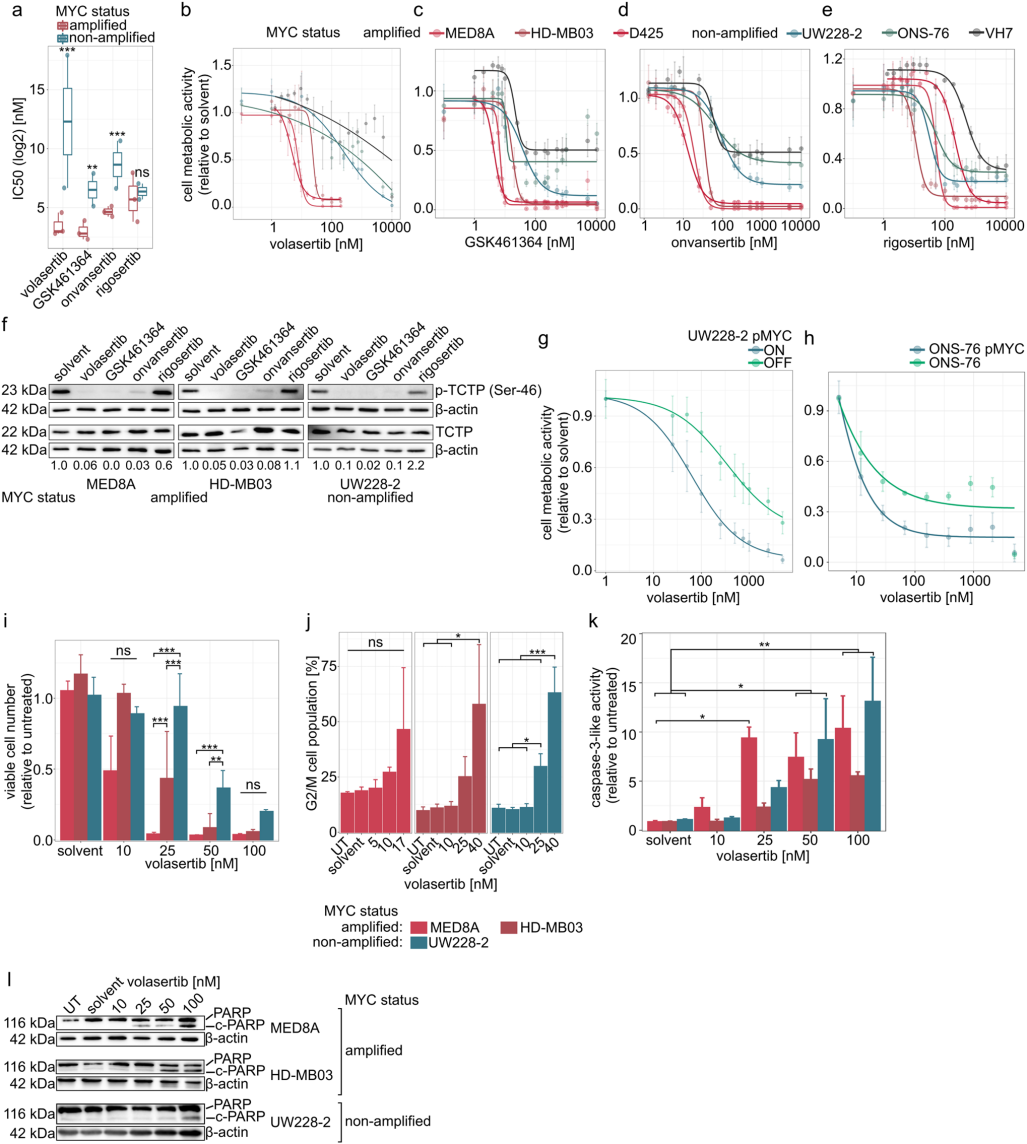
PLK1 inhibitors are selective to *MYC*-amplified MB cell line models. **a** Comparison of the IC50 (log2, nM) values of PLK1 inhibitors in *MYC*-amplified and non-amplified MB cell line models. **b**–**e** Dose–response plot of volasertib (**b**), GSK461364 (**c**), onvansertib (**d**) and rigosertib (**e**) based on metabolic activity in tested cell line models after 72 h treatment. **f** On-target effect of PLK1 inhibitors (volasertib, GSK461364, onvansertib and rigosertib, all 1 µM) in MB cell line models after 20 min treatment (quantification normalized to loading control and solvent). **g** Dose–response plot of volasertib based on metabolic activity in MYC-inducible UW228-2 cell line model after 72 h treatment. **h** Dose–response plot of volasertib based on metabolic activity in MYC-expressing ONS-76 cell line model after 72 h treatment. **i** Relative viable cell number in MB cell line models after volasertib treatment for 72 h. **j** percentage of single cell population in G2 phase in MB cell line models after volasertib treatment for 24 h. **k** Caspase-3-like activity after 24 h treatment with volasertib in MB cell line models. **l** PARP cleavage in *MYC*-amplified (MED8A, HD-MB03) and non-amplified (UW228-2) MB cell line models after 24 h volasertib treatment. *UT* untreated. *p < 0.05; **p < 0.01; ***p < 0.001; ns or no indication: not significant

Determination of single drug dose–response of several PLK1is (Suppl. Table 5) showed lower IC50s in *MYC*-amplified MB cells for all three ATP-competitive PLK1is (volasertib, GSK461364, onvansertib) (Fig. 2a–d, Suppl. Table 6), but not for the non-ATP-competitive dual activity PLK1 and PI3K inhibitor rigosertib (Fig. 2a, e, Suppl. Table 6). The activity of the ATP-competitive PLK1is on the fibroblast cell line VH7 was comparable to the activity on non-amplified MB cell lines, suggesting a presence of a therapeutic window in case of *MYC*-amplified MB. A loss of phosphorylation of the PLK1 downstream target TCTP confirmed the on-target-effect for all PLK1is except for rigosertib (Fig. 2f), which was subsequently excluded from further analyses. The increased PLK1i potency and efficacy observed in *MYC*-amplified cell lines (Fig. 2b–d) was confirmed in artificial models (Fig. 2g, h) where the MYC protein was overexpressed (Suppl. Fig. 5).

We therefore conclude that *MYC*-amplified MB cell lines are more sensitive to PLK1is, and that *MYC*-amplification could represent a predictive biomarker for response to PLKi treatment.

### Validation of PLK1 and class I HDAC inhibition in *MYC*-amplified MB

The subsequent experiments focused on volasertib due to advanced clinical development (Suppl. Table 5). Volasertib treatment of MB cell lines decreased the number of viable cells significantly in a concentration and MYC status-dependent manner (Fig. 2i). As expected, a concentration-dependent cell cycle arrest at G2/M phase was detected, without dependence on MYC status (Fig. 2j). A concentration-dependent increase of the caspase-3-like activity was detected in all MB cell lines (Fig. 2k). Finally, *MYC*-amplified cells exhibited PARP cleavage upon volasertib treatment at lower concentrations compared to *MYC*-non-amplified cells (Fig. 2l, Suppl. Fig. 6). Thus, volasertib leads to a significant decrease in the number of viable cells and increased PARP cleavage in a *MYC*-dependent manner in MB cells.

In line with previously published data on single agent class I HDACi treatment of MB cells [10, 12], we observed a *MYC*-status-dependent reduction of metabolic activity in the UW228-2-*MYC*-inducible cell line model (Suppl. Fig. 7a), viable cell number (Suppl. Fig. 7b), an increase of subG0/G1 (Suppl. Fig. 7c), caspase-3-like activity, and PARP cleavage upon treatment of MB cells with entinostat (Suppl. Fig. 7d–f). This data again confirms a *MYC* status-dependent response of MB cells to class I HDAC inhibition.

### Synergistic interaction of the class I HDACi entinostat and PLK1 inhibitors in MB

**Fig. 3:**
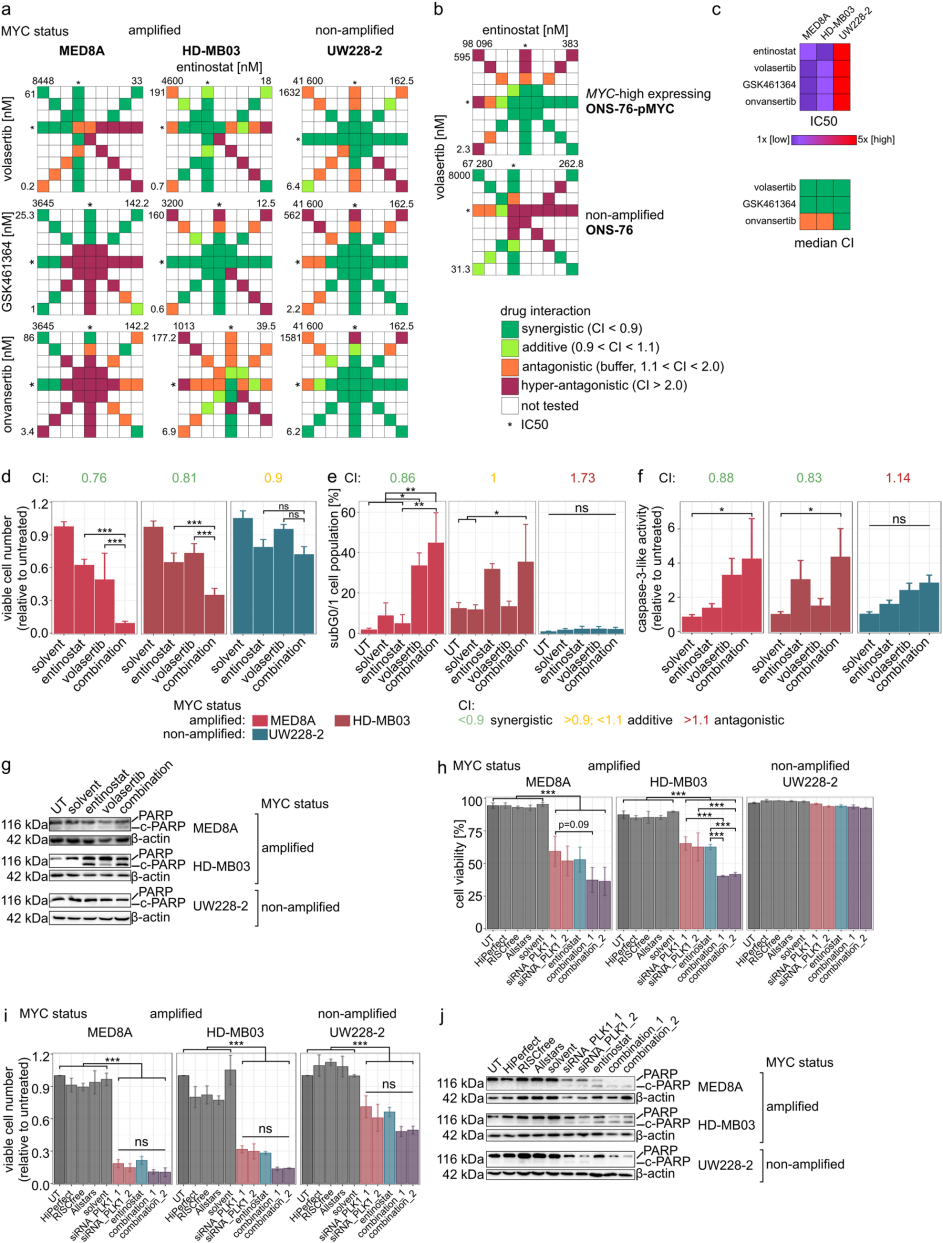
PLK1i and entinostat interact synergistically in MB. **a** PLK1i and entinostat (concentrations from 1/16x to 16x IC50) combination index (CI) tables in MB cell lines. **b** Volasertib and entinostat (concentrations from 1/16× to 16× IC50) combination index (CI) tables in ONS-76 wild-type cell line and ONS76 cell line transduced with MYC-expressing construct. **c** Entinostat and PLK1i IC50s used for combination evaluation heatmap in MED8A, HD-MB03 and UW228-2 and median combination indices (CIs) of entinostat and PLK1is in MB cell lines. **d** Relative viable cell number in MB cell line models after volasertib (MED8A: 10 nM, HD-MB03 and UW228-2: 15 nM), entinostat (500 nM) or combination treatment for 72 h. **e** Percentage of single cell population in subG0/1 fraction in MB cell line models after 48 h treatment. **f** Caspase-3-like activity after 24 h (MED8A) or 48 h (HD-MB03 and UW228-2) treatment with volasertib (MED8A: 10 nM, HD-MB03 and UW228-2: 15 nM), entinostat (1000 nM) or their combination in MB cell line models. **g** PARP cleavage in MB cell line models after 48 h volasertib (MED8A: 10 nM, HD-MB03 and UW228-2: 15 nM), entinostat (1000 nM) or combination treatment. **h** Percentage of viable cells in MB cell line models after 48 h siRNA-mediated PLK1 knock-down, entinostat (1000 nM) treatment or their combination. **i** Relative viable cell number in MB cell line models after 48 h PLK1 knock-down, entinostat (1000 nM) treatment or respective combination. **j** PARP cleavage in MB cell line models after 48 h siRNA-mediated PLK1 knock-down, entinostat (1000 nM) treatment or their combination. *UT* untreated. **p < 0.01; ***p < 0.001; ns or no indication: not significant. CI values in (**d)**, (**e)**, (**f)** were calculated using bliss independence model, green: synergistic, yellow: additive, red: antagonistic interaction

In a Chou–Talalay drug interaction model [30, 31]-based analysis, entinostat interacted with the PLK1is volasertib, GSK461364, and onvansertib (Fig. 3a). In particular, volasertib displayed synergism in all three models tested, while GSK461364 and onvansertib showed synergism in two out of three models (Fig. 3a, c). While synergism was detected in both *MYC*-amplified and non-amplified MB cell lines, the concentrations needed to achieve synergistic effects were lower and in a clinically achievable concentration range (comparable to the c_max_ reported by clinical trials [32, 33], Suppl. Table 5) in the *MYC* amplified background compared to the *MYC*-non-amplified background (Fig. 3c, Suppl. Fig. 8a–c). *MYC* expression in non-amplified ONS-76 led to synergistic interaction between entinostat and volasertib (Fig. 3b, Supp. Fig. 8d), confirming the contribution of *MYC* expression to PLK1i synergism with HDACi in a genetic model. Additive-to-synergistic interaction of entinostat and volasertib was observed in one out of two group 3 *MYC*-amplified PDX short-term cell cultures tested (Suppl. Fig. 8e).

### Validation of synergy of entinostat and volasertib in *MYC* amplified MB cells

The synergy of the combination of class I HDAC and PLK1 inhibition with entinostat and volasertib was validated by cell counts, cell cycle analysis, caspase-3-like activity assay, and PARP immunoblot. While cell cycle arrest was independent of *MYC* status (Fig. 2j), only *MYC*-amplified MB cells showed a synergistic reduction of the number of viable cells (Fig. 3d), a synergistic to additive increase in the subG0/G1 fraction (Fig. 3e), synergistic caspase-3-like activity (Fig. 3f), and increased PARP cleavage (Fig. 3g, Suppl. Fig. 9a, Suppl. Table 7). MYC-dependent effects of the combination treatments on cell viability and death were replicated in a genetic model combining siRNA-mediated PLK1 knock-down (Suppl. Fig. 9b) and entinostat treatment (Fig. 3h–j).

We conclude that class I HDAC and PLK1 inhibition with entinostat and volasertib is synergistic in most biological readouts at clinically achievable concentrations in the *MYC*-amplified MB cells.

### Mechanism of action of entinostat and volasertib as single agent and in combination

**Fig. 4:**
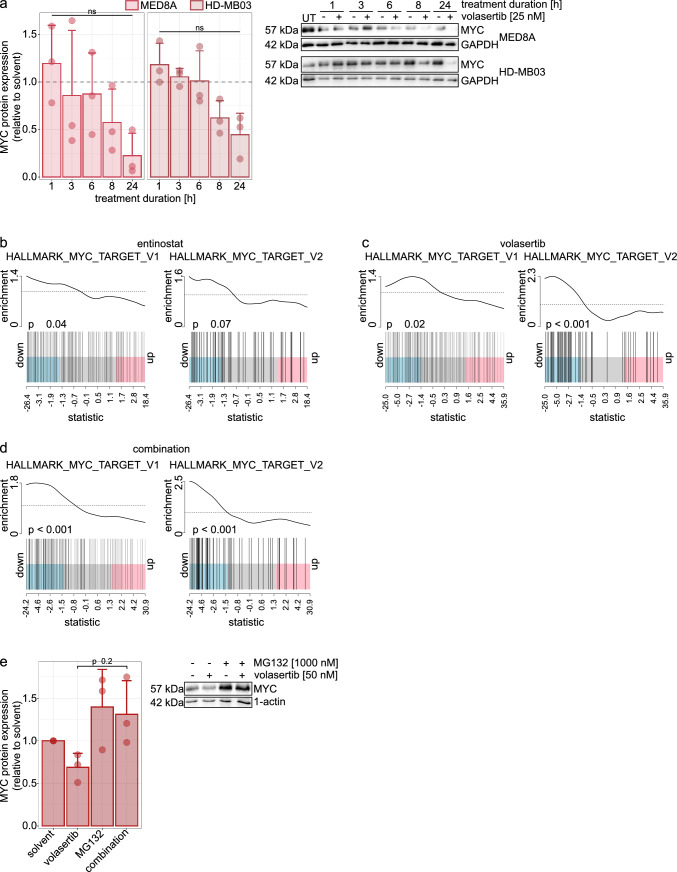
Volasertib as single agent and in combination with entinostat exert its’ activity via MYC. **a** Relative quantification and immunoblot example of MYC protein levels after 1, 3, 6, 8 and 24 h treatment with 25 nM volasertib in *MYC*-amplified MB cell lines (normalized to loading control and timepoint-matched solvent control). **b–d** Gene set enrichment analysis of HALLMARK_MYC_TARGET_V1 and V2 [26] gene sets after entinostat (5 µM, **b**), volasertib (1 µM, **c**) and combination (**d**) treatment for 6 h. **e** relative quantification and immunoblot example of MYC protein levels after 3 h treatment with 50 nM volasertib, 1000 nM MG132 or their combination in *MYC*-amplified MB cell line HD-MB03 (normalized to loading control and solvent). *UT* untreated. ns or no indication: not significant

Our data indicates *MYC* amplification as a possible biomarker for the treatment with class I HDACi and PLK1i. We therefore investigated MYC´s role in the cellular response to the combination treatment. It has been shown that MYC target genes are downregulated upon entinostat treatment [12]. As MYC protein expression was reduced upon volasertib treatment (Fig. 4a), we examined the effect on MYC target genes in the combination treatment. Gene expression profiling of *MYC*-amplified HD-MB03 cells treated for 6 h with entinostat, volasertib, or the combination, revealed a significant down-regulation of MYC target gene sets (HALLMARK_MYC_TARGET_V1 and V2 [26]) in all three conditions (Fig. 4b–d). The short time point was chosen to minimize secondary effects. The reduction of MYC target gene expression points to a direct effect of entinostat and volasertib on the transcriptional activity of MYC. MYC protein levels were rescued by MG132 proteasome inhibitor treatment (Fig. 4e), suggesting proteasomal degradation of MYC upon volasertib treatment, in line with published data [34]. Taken together, this data indicates entinostat and volasertib exert their activity via MYC protein transcriptional activity, both as single drugs and in combination and PLK1 inhibition targets MYC for proteasomal degradation in MB cells.

### Entinostat and volasertib combination evaluation in vivo

**Fig. 5:**
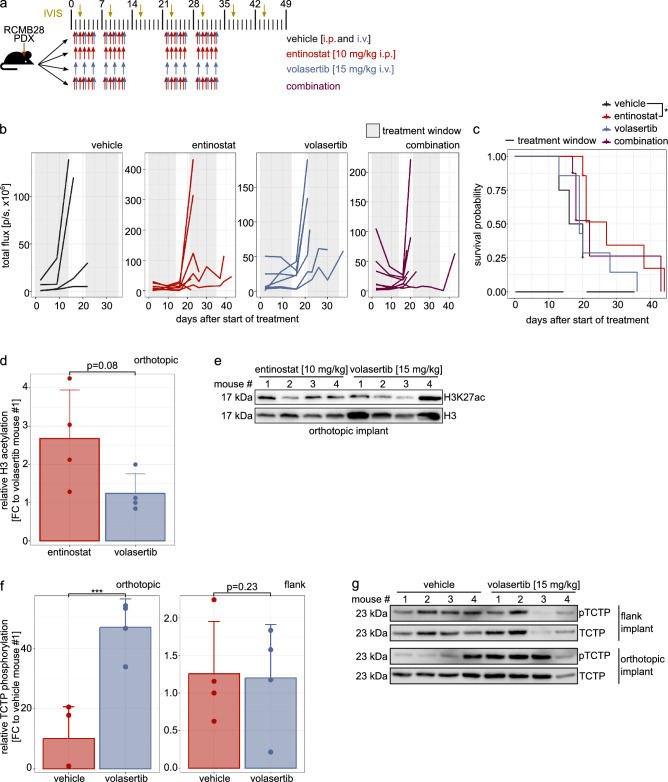
Entinostat treatment prolongs tumor-bearing mouse survival. **a** Treatment and monitoring scheme. **b** Tumor volume during and after vehicle, entinostat, volasertib and combination treatment. **c** Tumor-bearing animal survival analysis comparing mice treated with vehicle, entinostat, volasertib or combination. **d** Relative quantification of H3K27ac levels after entinostat and volasertib treatment in orthotopic tumor (normalized to loading control and H3 levels). **e** Immunoblot image of H3K27ac and H3 levels after entinostat and volasertib treatment in orthotopic tumor. **f** Relative quantification of pTCTP levels in orthotopic or flank tumors after volasertib treatment (normalized to loading control and TCTP levels). **g** Immunoblot image of pTCTP and TCTP levels in orthotopic or flank tumors after volasertib treatment. *p < 0.05, ***p < 0.001, ns or no indication: not significant

Entinostat and volasertib single treatment and combination were evaluated in an orthotopic PDX model (group 3 *MYC*-amplified MB RCMB28). Tumor-bearing mice were treated for four weeks, with one week interruption (Fig. 5a) and monitored for tumor growth and survival.

Weight loss was not associated with treatment (Suppl. Fig. 10a–c). During the first two weeks of treatment, we observed a reduction in tumor volume in the combination-treated mice compared to other groups, and reduced growth in the single drug treatments (Fig. 5b, Suppl. Fig. 10d). However, upon pause of treatment in week three, tumors started to quickly regrow. Overall, no volasertib treatment-associated effect on survival was observed, neither in the single treatment, nor in the combination group, whereas entinostat single treatment significantly prolonged survival compared to vehicle control (Fig. 5c). Assessment of H3 acetylation showed on-target activity of entinostat, indicating blood–brain barrier (BBB) penetration (Fig. 5d, e). However, no volasertib on-target effect was observed in vivo, neither in orthotopic nor in flank implants of HD-MB03 cells (Fig. 5f, g).

## Discussion

The oncoprotein MYC is associated with poor clinical outcome in several cancer entities, including CNS tumors. MBs with *MYC* amplification often present a phenotype of MYC addiction, thus making MYC a very attractive target for therapy. However, MYC is difficult to target directly. Therefore, other strategies, including targeting the transcriptional and post-translational regulation of MYC, have been used to target MYC-addicted tumors [8]. We and others have previously shown that *MYC*-amplified MB cells are more susceptible to HDACi treatment compared to *MYC*-non-amplified cells [10, 11]. However, moderate efficacy and dose-limiting toxicities, especially in pan-HDAC inhibitors, have been reported [35]. Synergistic combination of HDACi with other drugs, such as standard chemotherapies [36] or targeted compounds, could potentially increase the observed anti-tumor effect and, by virtue of dose-reduction, reduce the adverse effects of HDACi treatment. We proposed PLK1i, e.g. volasertib, as a potential partner for combination therapy with a class I HDAC inhibitors, e.g. entinostat.

The MYC-target gene *PLK1* is a well-known cell cycle regulator and oncogene, implicated in the development of several cancer types [37]. PLK1 regulates MYC stability via the E3 ubiquitin ligase FBXW7. FBXW7 mediates MYC turnover by promoting ubiquitin-mediated proteasomal degradation. PLK1 phosphorylates FBXW7, marking it for ubiquitination and subsequent degradation, thus stabilizing MYC indirectly, and closing MYC and PLK1 in a feedback loop [34]. PLK1 has been shown to be a valid target in *MYC*-driven cells such as lymphoma [38], glioma [39], and medulloblastoma, where onvansertib sensitized tumors to radiotherapy [40], and pediatric malignancies synergizing with vincristine [41]. In line with previous reports [34, 42], we now show that PLK1i treatment leads to loss of MYC protein in MB. In addition, we show *PLK1* overexpression in MYC-driven MB subgroups, its downregulation upon class I HDAC inhibition, and a strong suppression of MYC target gene expression after combination treatment. The latter results are in line with our previously published data showing that class I HDAC inhibition reduces MYC transcriptional activity. However, as opposed to PLK1i degrading MYC itself, class I HDACi leads to stabilization of transcriptionally inactive MYC [12], making it complementary to the mechanism of action of PLK1i, and both resulting in the same net effect of suppression of MYC transcriptional activity (Suppl. Fig. 11).

The sensitivity for HDACis or PLK1is was higher in *MYC*-amplified MB cell lines. PLK1 inhibitors have been previously employed in medulloblastoma, showing promise as radiosensitizers [40] and in combination with BET inhibitors [43, 44]. Moreover, HDACs and PLK1 have been previously reported to be associated with MYC [10]. We therefore hypothesized that the interaction has a potential to be synergistic. PLK1is were indeed shown to synergize with pan-HDAC inhibitors in hematological malignancies [42], but synergism of PLK1i and HDACi in MB has not been previously described. We here report that entinostat and PLK1is interact synergistically in MB cells in clinically achievable concentrations [32, 33] in *MYC*-amplified cells only. Several PLK1is (e.g. volasertib, GSK461364 and rigosertib), have been tested in clinical trials, but similar to HDACis, only showed moderate single agent efficacy, with substantial toxicities [45]. In line with previous studies in non-Hodgkin’s lymphoma [42], our results suggest synergistic interaction between class I HDACi and PLK1i in *MYC*-amplified MB cell lines in vitro .

While our data in orthotopic PDX confirmed the effectivity of class I HDACi in *MYC*-amplified MB, we did not observe an anti-tumor effect of volasertib alone, nor a synergistic effect on tumor size or survival in the combination. While the lack of volasertib effect on intracranial PDX tumor growth could be attributed to the insufficient BBB penetrance which was previously suggested [46], volasertib did not reach the target in flank tumors as well, indicating a more general poor PD activity of volasertib in mice. Alternative PLK1 inhibitors with more favorable PD activity such as GSK461364, shown to have an effect on GBM in mice [47], will need to be tested in combination with class I HDACi in vivo.

In summary, we conclude that PLK1is act synergistically with class I HDACis in MYC-driven MB cells. Single treatment with both entinostat and PLK1 ATP-competitive inhibitors shows selective sensitivity of *MYC*-amplified MB cells. And while we observe some anti-cancer effects of PLK1is as single agents in *MYC*-non-amplified background, it is less significant compared to *MYC*-amplified cells, possibly due to moderate expression of and dependence on of MYC and/or PLK1. The class I HDACi entinostat and PLK1is show synergistic interaction in MB cells in clinically achievable concentrations only in MYC-driven tumors. The mechanism of action is executed via MYC transcriptional activity. The HDACi entinostat significantly prolonged survival of PDX tumor-bearing animals, while the effect of PLK1i on orthotopic PDX MB warrants further investigation with more suitable, i.e., BBB penetrant PLK1is. The clear biomarker elucidation and activity at clinically achievable drug concentrations of the combination indicate a strong translational potential for further clinical development in MYC-driven MB.

## Supplementary Information

Below is the link to the electronic supplementary material.

Supplementary material 1 (PDF 1417.1 kb)

Supplementary material 2 (PDF 1371.2 kb)

Supplementary material 3 (PDF 164.4 kb)
