# Supplementary material for: Class I HDAC inhibitor entinostat synergizes with PLK1 inhibitors in MYC-amplified medulloblastoma cells
Source: J Neurooncol. 2023 May 15;163(1):143–58. doi: 10.1007/s11060-023-04319-1 (PMC10232604; doi:10.1007/s11060-023-04319-1)
Supplement: Supplementary file 1 — Supplementary material 1 (PDF 1417.1 kb) [file 11060_2023_4319_MOESM1_ESM.pdf]

Class I HDAC inhibitor entinostat synergizes with PLK1 inhibitors in *MYC*-amplified medulloblastoma cells

Gintvile Valinciute[1,2,15], Jonas Ecker[1-3], Florian Selt[1-3], Thomas Hielscher[5], Romain Sigaud[1,2], Johannes Ridinger[1,2], Venu Thatikonda[1,13,#], Charlotte Gatzweiler[1,2], Sarah Robinson[15], Julie Talbot[6,7], Flavia Bernardi[6,7], Daniel Picard[8,9], Mirjam Blattner-Johnson[1,10], Simone Schmid[11,12], David T. Jones[1,10], Cornelis M. van Tilburg[1-3], David Capper[11,12], Marcel Kool[1,13,14], Marc Remke[8,9], Ina Oehme[1,2], Stefan M. Pfister[1,3,13], Martine F. Roussel[15], Olivier Ayrault[6,7], Olaf Witt[1-3], Till Milde[1-3,*]

1 Hopp Children's Cancer Center Heidelberg (KiTZ), Heidelberg, Germany

2 Clinical Cooperation Unit Pediatric Oncology, German Cancer Research Center (DKFZ) and German Consortium for Translational Research (DKTK), Heidelberg, Germany

3 KiTZ Clinical Trial Unit (ZIPO), Department of Pediatric Hematology and Oncology, Heidelberg University Hospital, Heidelberg, Germany

5 Division of Biostatistics, German Cancer Research Center (DKFZ), Heidelberg, Germany

6 Institut Curie, PSL Research University, CNRS UMR, INSERM, Orsay, France

7 Université Paris Sud, Université Paris-Saclay, CNRS UMR 3347, INSERM U1021, Orsay, France

8 Department of Pediatric Oncology, Hematology and Clinical Immunology, Medical Faculty, University Hospital Düsseldorf, Düsseldorf, Germany

9 Department of Pediatric Neuro-Oncogenomics, German Cancer Research Center (DKFZ), Heidelberg, Germany; and German Cancer Consortium (DKTK), partner site Essen/ Düsseldorf, Düsseldorf, Germany

10 Division of Pediatric Glioma Research, German Cancer Research Center (DKFZ), Heidelberg, Germany

11 Department of Neuropathology, Charité - Universitätsmedizin Berlin, Germany

12 DKTK Partner Site, Berlin, Germany

13 Division of Pediatric Neurooncology, German Cancer Research Center (DKFZ) and German Consortium for Translational Research (DKTK), Heidelberg, Germany

14 Princess Máxima Center for Pediatric Oncology, Utrecht, the Netherlands

15 Department of Tumor Cell Biology, St. Jude Children's Research Hospital, Memphis, TN, USA

# Present address: Global Computational Biology and Digital Sciences, Boehringer Ingelheim RCV GmbH; Co KG, Doktor-Boehringer-Gasse 5-11, 1120 Vienna, Austria

* Corresponding author: Till Milde, Hopp Children's Cancer Center Heidelberg (KiTZ), CCU Pediatric Oncology B310, German Cancer Research Center (DKFZ), Im Neuenheimer Feld 280, 69120 Heidelberg, Germany; Tel.: +49 6221 42 3574; email: t.milde@kitz-heidelberg.de

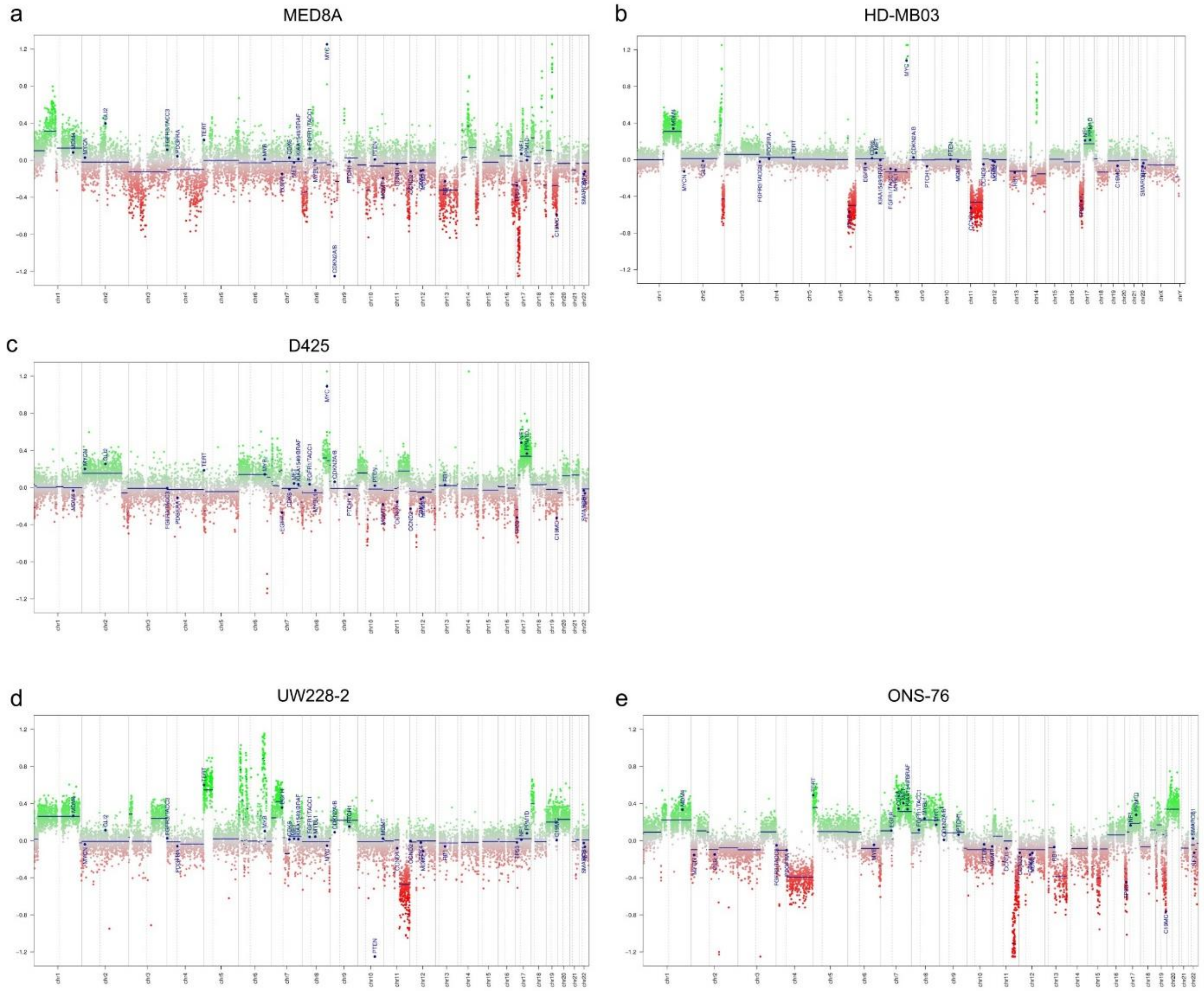

Supplementary figure 1. DNA methylation array-derived copy number variation (CNV) plots generated in cell lines used in this study. a – MED8A, b – HD-MB03, c – D425, d – UW228-2, e – ONS-76.

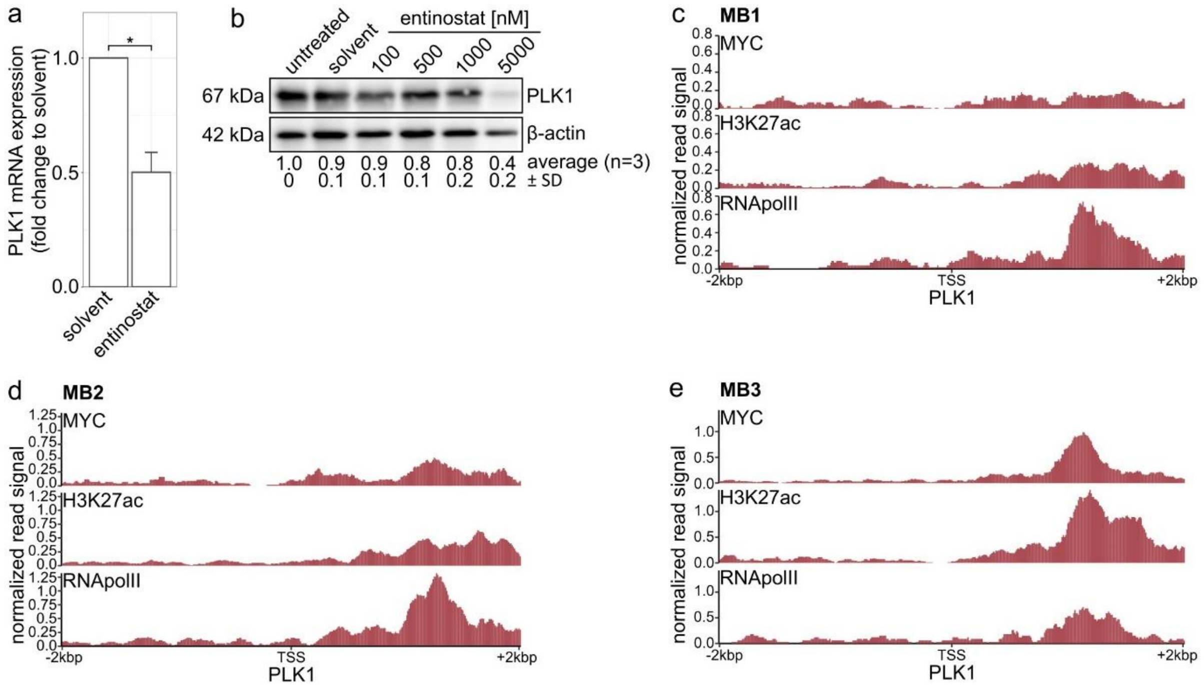

Supplementary figure 2. PLK1 target validation; MYC, H3K27ac and RNAPolII ChIP signal in MB tumors. a – Relative PLK1 mRNA expression measured by qRT-PCR in HD-MB03 MB cells treated with 5 µM entinostat for 6 h (t-test, *p < 0.05). b – PLK1 protein levels after 48-hour entinostat treatment, quantified,normalized to loading control and untreated sample and averaged from three independent biological replicates (±SD). c-e – MYC, H3K27ac and RNAPolII ChIP signal on PLK1 promoter in 3 primary MB tumors.

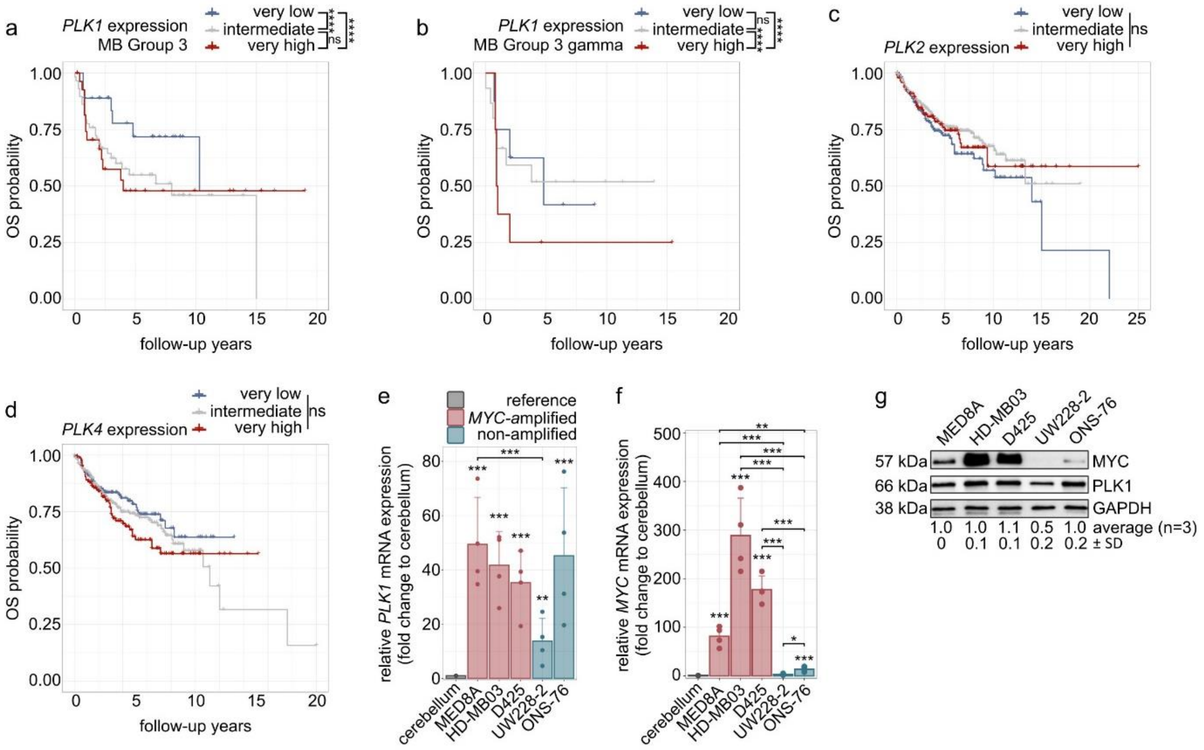

Supplementary figure 3. OS differences in PLK2 and PLK4 expression groups and PLK1, MYC expression in MB cell lines. a – Overall survival (OS) differences between *PLK1* mRNA high (above Q3), low (below Q1) and intermediate expressing patients in group 3 subgroup of MB (Cavalli[1]; Log Rank test). b – Overall survival (OS) differences between *PLK1* mRNA high (above Q3), low (below Q1) and intermediate expressing patients in group 3 γ subtype associated to MYC amplification (Cavalli[1]; Log Rank test). c – Overall survival (OS) differences between *PLK2* mRNA high (above Q3), low (below Q1) and intermediate expressing tumor patients (Cavalli (n=763)[1]; Log Rank test). d – Overall survival (OS) differences between *PLK4* mRNA high (above Q3), low (below Q1) and intermediate expressing tumor patients (Cavalli (n=763)[1]; Log Rank test). e – Relative *PLK1* mRNA expression in MB cell line models (red: *MYC*-amplified, blue: non-amplified). f – Relative *MYC* mRNA expression in MB cell line models (red: *MYC*-amplified, blue: non-amplified). g – PLK1 and MYC protein levels in MB cell lines. *p < 0.05; **p < 0.01; ***p < 0.001; ns or no indication: not significant.

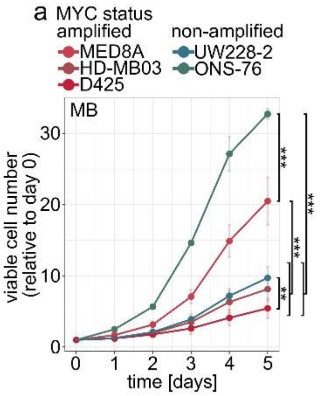

Supplementary figure 4. Cell growth curves in MB model cell lines. a – Growth curves of MB cell line models. **p < 0.01; ***p < 0.001; ns or no indication: not significant.

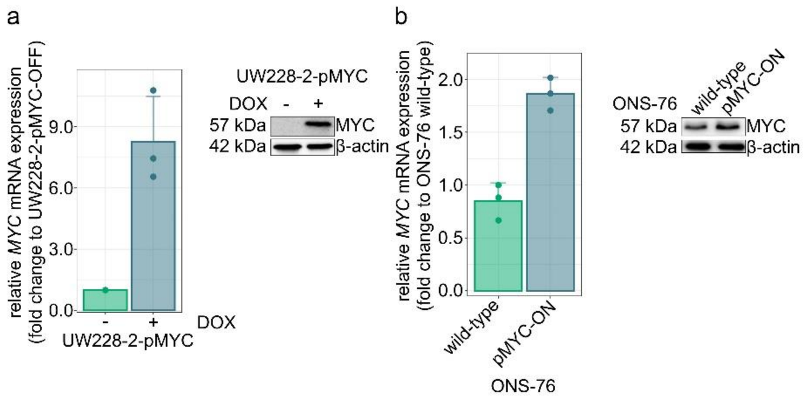

Supplementary figure 5. UW228-2 and ONS-76 transduced with MYC-expressing constructs. a – Relative *MYC* mRNA expression (left) and MYC protein expression (right) in UW228-2-MYC-inducible cell line model in OFF and ON modes. b – Relative *MYC* mRNA expression (left) and MYC protein expression (right) in ONS-76 wild-type and expressing MYC construct cell line models.

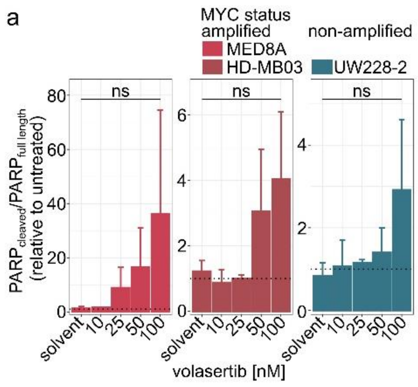

Supplementary figure 6. PARP cleavage in volasertib-treated MB cells. a – Proportion of cleaved and full-length PARP after 24 h treatment with volasertib in MB cell line models (quantification normalized to loading control and untreated sample). ns: not significant.

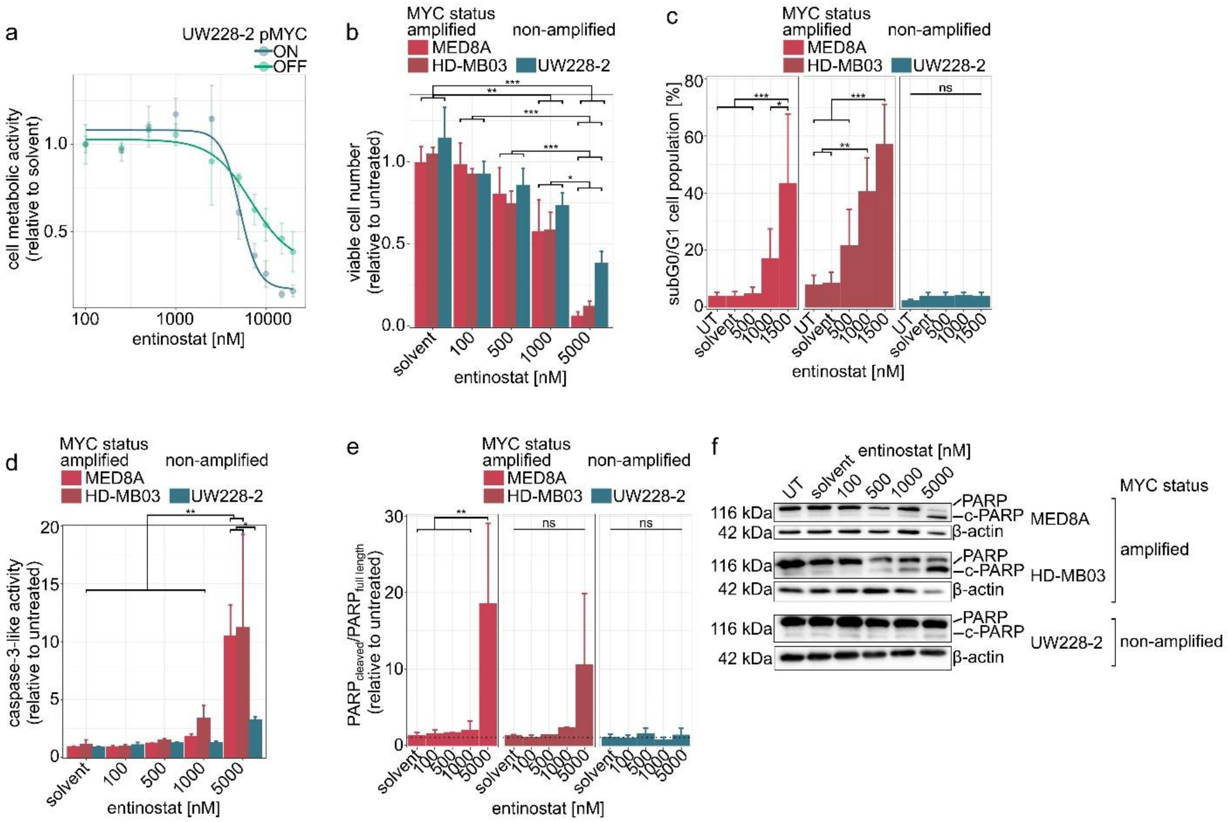

Supplementary figure 7. Entinostat in MB cell line models. a – Entinostat drug-response curves in UW228-2-MYC-inducible cell line in OFF and ON modes after 72 h of treatment. b – Relative viable cell number in MB cell line models after 72 h entinostat treatment. c – Percentage of single cell population in the subG0/1 cell cycle fraction in MB cell line models after entinostat treatment for 48 h. d – Caspase-3-like activity after 48 h treatment with entinostat in MB cell line models. e – Proportion of cleaved and full-length PARP after 48 h treatment with entinostat in MB cell line models (quantification normalized to loading control and untreated sample). f – PARP cleavage in MB cell line models after 48 h entinostat treatment. UT: untreated. *$p < 0.05$; **$p < 0.01$; ***$p < 0.001$; ns or no indication: not significant.

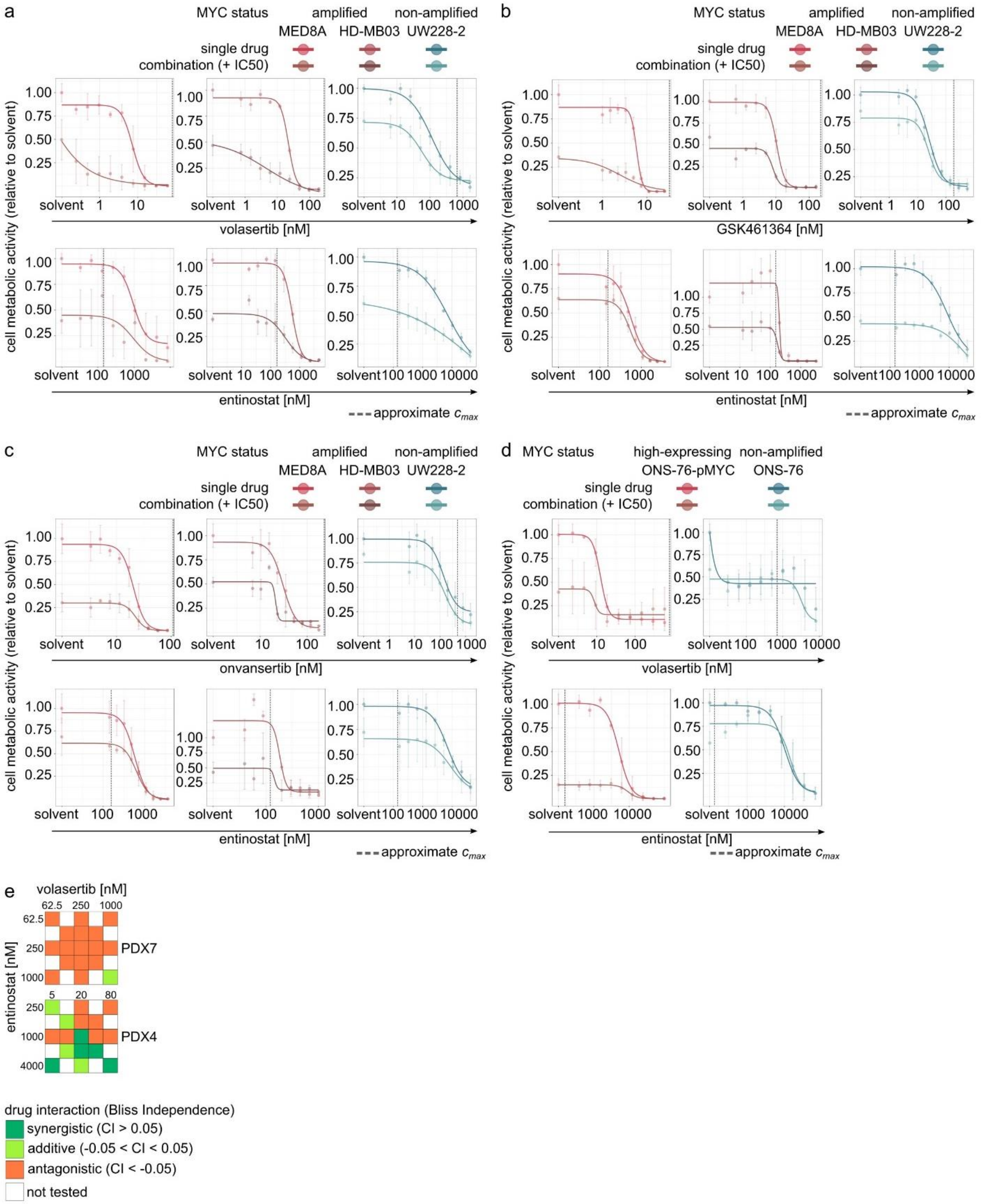

Supplementary figure 8. Interaction of entinostat and PLK1 inhibitors. a – Dose-response curves with volasertib (upper panel) and entinostat (lower panel) single drugs compared to combination in MB cell line models. b – Dose-response curves with GSK461364 (upper panel) and

entinostat single drugs or in combination in MB cell line models. c – Dose-response curves with onvansertib (upper panel) and entinostat single drugs or in combination in MB cell line models. d – Dose-response curves with volasertib (upper panel) and entinostat (lower panel) single drugs compared to combination in ONS-76 wild-type cell line and ONS76 cell line transduced with MYC-expressing construct. e – Volasertib and entinostat Bliss independence model-calculated CI table in short-term PDX cell culture in two MYC amplified group 3 MB PDX models. c_{max}: maximum plasma concentration.

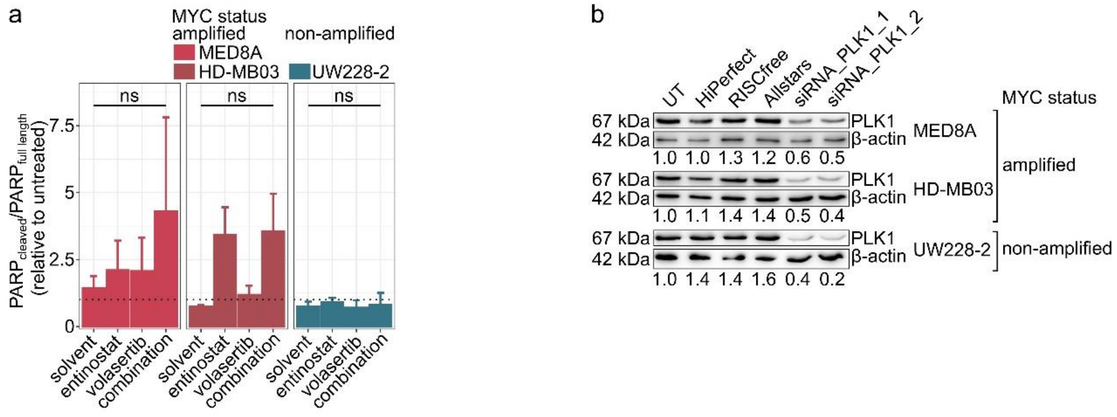

Supplementary figure 9. Volasertib and entinostat interaction in MB cell line models. a – Proportion of cleaved and full-length PARP after 48 h treatment with entinostat (1000 nM), volasertib (MED8A: 10 nM, HD-MB03 and UW228-2: 15 nM) or their combination in MB cell line models (quantification normalized to loading control and untreated sample). B – PLK1 knock-down efficiency after 24 h in MB cell line models (quantification is the average of 3 biological replicates and normalized to loading control and untreated sample). UT: untreated. ns: not significant.

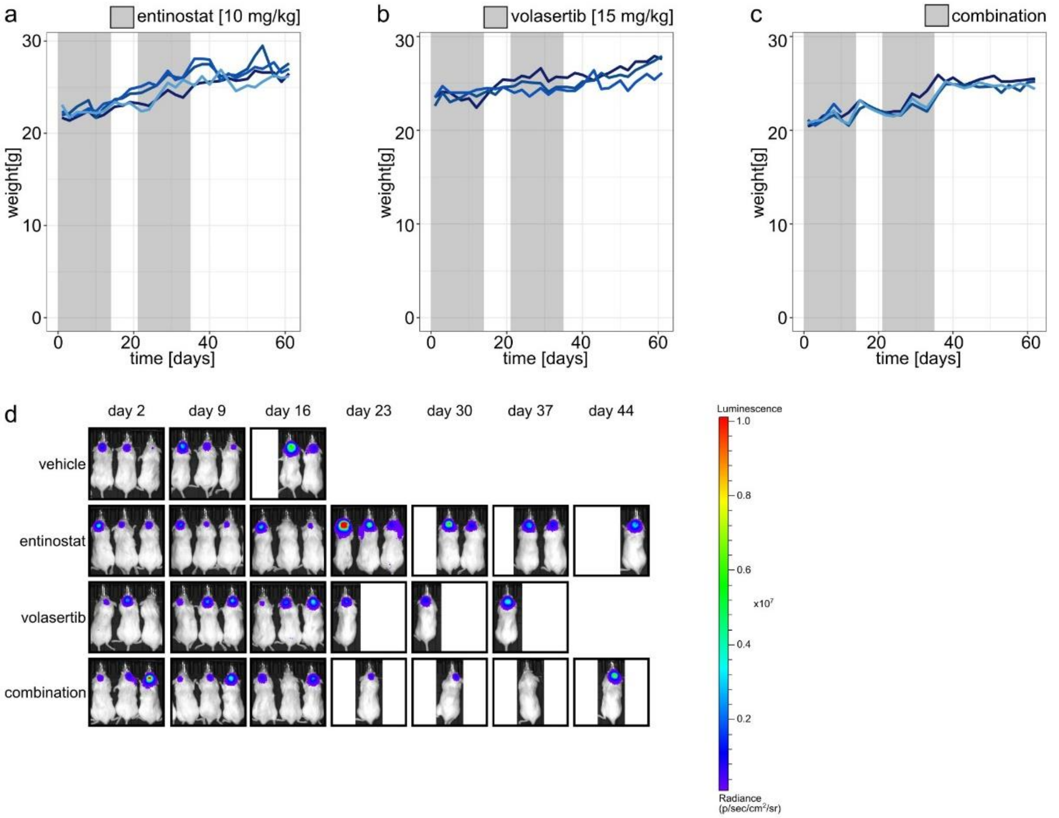

Supplementary figure 10. Volasertib and entinostat treatment in orthotopic PDX animals. a, b, c – healthy mouse weight during and after treatment with entinostat (a, n=4, 10 mg/kg), volasertib (b, n=3, 15 mg/kg) or their combination (c, n=3). d – Representative examples of mouse tumor sizes during and after treatment with vehicle, entinostat (10 m/kg), volasertib (15 mg/kg) or combination.

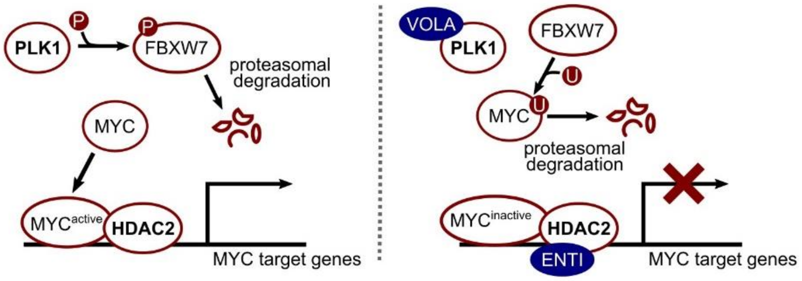

Supplementary figure 11. Proposed mechanism of action model of entinostat and volasertib combination. VOLA: volasertib; ENTI: entinostat; P: phosphorylation; U: ubiquitinylation.

References

1. Cavalli FMG, Remke M, Rampasek L, Peacock J, Shih DJH, Luu B, Garzia L, Torchia J, Nor C, Morrissy AS, Agnihotri S, Thompson YY, Kuzan-Fischer CM, Farooq H, Isaev K, Daniels C, Cho BK, Kim SK, Wang KC, Lee JY, Grajkowska WA, Perek-Polnik M, Vasiljevic A, Faure-Conter C, Jouvet A, Giannini C, Nageswara Rao AA, Li KKW, Ng HK, Eberhart CG, Pollack IF, Hamilton RL, Gillespie GY, Olson JM, Leary S, Weiss WA, Lach B, Chambless LB, Thompson RC, Cooper MK, Vibhakar R, Hauser P, van Veelen MC, Kros JM, French PJ, Ra YS, Kumabe T, Lopez-Aguilar E, Zitterbart K, Sterba J, Finocchiaro G, Massimino M, Van Meir EG, Osuka S, Shofuda T, Klekner A, Zollo M, Leonard JR, Rubin JB, Jabado N, Albrecht S, Mora J, Van Meter TE, Jung S, Moore AS, Hallahan AR, Chan JA, Tirapelli DPC, Carlotti CG, Fouladi M, Pimentel J, Faria CC, Saad AG, Massimi L, Liau LM, Wheeler H, Nakamura H, Elbabaa SK, Perezpena-Diazconti M, Chico Ponce de Leon F, Robinson S, Zapotocky M, Lassaletta A, Huang A, Hawkins CE, Tabori U, Bouffet E, Bartels U, Dirks PB, Rutka JT, Bader GD, Reimand J, Goldenberg A, Ramaswamy V, Taylor MD (2017) Intertumoral Heterogeneity within Medulloblastoma Subgroups. Cancer Cell 31: 737-754 e736 doi:10.1016/j.ccell.2017.05.005
