# Supplementary material for: Class I HDAC inhibitor entinostat synergizes with PLK1 inhibitors in MYC-amplified medulloblastoma cells
Source: J Neurooncol. 2023 May 15;163(1):143–58. doi: 10.1007/s11060-023-04319-1 (PMC10232604; doi:10.1007/s11060-023-04319-1)
Supplement: Supplementary file 2 — Supplementary material 2 (PDF 1371.2 kb) [file 11060_2023_4319_MOESM2_ESM.pdf]

Supplementary table 1. Inhibitors used for the assays in vitro.

Target	Inhibitor	Synonym	Solvent	Cat. No.	Manufacturer
Class I HDAC	Entinostat	MS-275	DMSO	S1053	Biozol Diagnostica Vertrieb GmbH, Eching, Germany
PLK1	Volasertib	BI-6727	DMSO	S2235	Biozol Diagnostica Vertrieb GmbH, Eching, Germany
	GSK461264	-	DMSO	S2193	Biozol Diagnostica Vertrieb GmbH, Eching, Germany
	Rigosertib	ON-01910	DMSO	S1362	Biozol Diagnostica Vertrieb GmbH, Eching, Germany
	Onvansertib	NMS-P937	DMSO	T6247-1ml-TM	Biocat GmbH, Heidelberg, Germany
Proteasome	MG132	-	DMSO	-	Biomol GmbH, Hamburg, Germany

Supplementary table 2. Primers used for qRT-PCR assay

Target	Name	Cat. No.	Company
ACTB	Hs_ACTB_2_SG QuantiTect Primer Assay	QT01680476	Qiagen
GAPDH	Hs_GAPDH_2_SG QuantiTect Primer Assay	QT01192646	Qiagen
MYC	Hs_MYC_1_SG QuantiTect Primer Assay	QT00035406	Qiagen
MYCN	Hs_MYCN_1_SG QuantiTect Primer Assay	QT00201404	Qiagen
PLK1	Hs_PLK1_1_SG QuantiTect Primer Assay	QT00049749	Qiagen

Supplementary table 3. Antibodies used for immunoblotting

Target	Host	Dilution	Manufacturer	Cat. No.
ACTB	Mouse	1:10000	Sigma	A5441
GAPDH	Mouse	1:10000	EMD Millipore	MAB374
MYC	Rabbit	1:10000	Abcam	ab32072
PARP	Rabbit	1:5000	Cell Signaling	9532S
PLK1	Mouse	1:5000	Abcam	ab17056
p-TCTP (Ser-46)	Rabbit	1:1000	Cell Signaling	5251S
TCTP	Rabbit	1:5000	Abcam	ab133568
ac-H3 (Lys-27)	Rabbit	1:1000	Abcam	ab4729
H3	Rabbit	1:5000	Cell Signaling	4499S

Supplementary Table 4. Significantly regulated genes from HALLMARK_MYC_TARGET_V1 and V2 upon entinostat treatment (5 μM; 6 hours).

Gene	LogFC	Adj.P.Val	Encoded protein	Biological process	Druggable (y/n)	Clinical development
**CCNA2**	-0.5509	0.00891	Cyclin-A2	Cell cycle; mitosis	n	NA
**PLK4**	-0.3791	0.01382	Polo-like kinase 4	Cell cycle	y	P1/P2, in combination with immunotherapy
**CDC20**	-0.4651	0.01569	Cell division cycle protein 20 homolog	Cell cycle; mitosis	n	NA
**NOP16**	-0.5067	0.01701	Nucleolar protein 16	Ribosome biogenesis	n	NA
**MPHOSPH10**	-0.3455	0.01886	U3 small nucleolar ribonucleoprotein protein MPP10	Ribosome biogenesis	n	NA
**SLC19A1**	-0.4795	0.01968	Reduced folate transporter	Intra/Inter-cellular transport	n	NA
**WDR74**	-0.4001	0.02107	WD repeat-containing protein 74	Ribosome biogenesis	n	NA
**PLK1**	-0.5207	0.0272	Polo-like kinase 1	Cell cycle; mitosis	y	Up to P3
**SRSF7**	-0.5639	0.03212	Serine/arginine-rich splicing factor 7	RNA processing	n	NA
**CUL1**	-0.3478	0.03217	Cullin-1	Protein ubiquitination	y	Pre-clinical
**SRSF1**	-0.7527	0.03343	Serine/arginine-rich splicing factor 1	RNA processing	n	NA
**CTPS1**	-0.3708	0.03418	CTP synthase 1	Pyrimidine biosynthesis	n	NA
**CSTF2**	-0.3669	0.03637	Cleavage stimulation factor subunit 2	RNA processing	n	NA
**NIP7**	-0.3553	0.03859	60S ribosome subunit biogenesis protein NIP7 homolog	Ribosome biogenesis	n	NA
**NDUFAF4**	-0.2742	0.03959	NADH dehydrogenase [ubiquinone] 1 alpha subcomplex assembly factor 4	Respiratory chain assembly	n	NA
**SUPV3L1**	-0.3021	0.04335	ATP-dependent RNA helicase SUPV3L1, mitochondrial	Mitochondrial RNA metabolism	n	NA
**GRWD1**	-0.2924	0.04508	Glutamate-rich WD repeat-containing protein 1	DNA replication	n	NA

Supplementary table 5. PLK1 inhibitors currently available for pre-clinical and clinical testing and tested in this study.

Inhibitor	Synonyms	Clinical development	Target	Selectivity [nM]*				Ki for PLK1 [nM]	c_{max}		Blood-brain barrier penetrance
				PLK1	PLK2	PLK3	Other targets		[ng/mL]/mg	Clinical trial	
**Volasertib**	BI 6727	Phase 3 (active, not recruiting)	ATP-binding domain	0.87	5	56	ND	ND	1.17-2.4	NCT01662505; NCT00969761; NCT01206816	+
**Rigosertib**	ON-01910	Phase 3 (recruiting)	Non-competitive	9	260	>10000	PI3K	ND	1.4-8.47	NCT01168011	+
**Onvansertib**	NMS-P937; NMS1286937	Phase 2 (recruiting)	ATP-binding domain	2	>10000	>10000	ND	ND	7.4-11.25*	NCT01014429	ND
**GSK461364**		Phase 1 (completed)	ATP-binding domain	2.2	ND	ND	ND	<0.5	1-1.82	NCT00536835	+++

* c_{max} shown in [ng/mL]/[mg/m^2]

ND = no data

Data collected from ChEMBL and PubChem databases

Supplementary table 6. IC50 values of single agent treatments in tested cell line models.

Entity			MB								Non-transformed human fibroblast
Cell line			MED8A	HD-MB03	D425	UW228-2	ONS-76	UW228-2 pMYC ON	UW228-2 pMYC OFF	ONS-76 pMYC	VH7
MYC(N) **amp**			*MYC*	*MYC*	*MYC*	-	-	*MYC*	-	*MYC*	-
IC50 [nM]	HDACi	Entinostat	730	580	322.6	2600	25×10^6	5226	6880	6131	6544
	PLK1i	Volasertib	8	24	5	102	25×10^4	68.1	350	37.2	--*
		GSK1461364	7	15	4.1	35	239				--*
		Onvansertib	19	35	12.9	99	1628				428.9
		Rigosertib	52	14	216.6	52	132				506.8

* impossible to calculate with non-restricted 5-parameter model

Supplementary table 7. Bliss independence model-calculated scores and combination indeces (CI) of entinostat and volasertib combination.

Assay	Cell_line	MYC status	Combination effect	Bliss	CI
Viable cell number	MED8A	Amplified	0.911375	0.697192	0.764988
	HD-MB03	Amplified	0.65316	0.528054	0.80846
	UW228-2	Non-amplified	0.281187	0.253744	0.902404
Flow cytometry [subG0/1 phase; 72 h]	MED8A	Amplified	0.894	0.7718	0.8633
	HD-MB03	Amplified	0.1154	0.1158	1.0041
	UW228-2	Non-amplified	0.0576	0.0996	1.7285
Caspase-3-like activity	MED8A	Amplified	0.846286	0.748924	0.884954
	HD-MB03	Amplified	0.867961	0.721038	0.830726
	UW228-2	Non-amplified	0.564465	0.642315	1.137919

Synergistic interaction (green): CI 0.764988, 0.80846, 0.8633, 0.884954, 0.830726
Additive interaction (yellow): CI 0.902404, 1.0041
Buffer-antagonistic interaction (red): CI 1.7285, 1.137919
