# Supplementary material for: Class I HDAC inhibitor entinostat synergizes with PLK1 inhibitors in MYC-amplified medulloblastoma cells
Source: J Neurooncol. 2023 May 15;163(1):143–58. doi: 10.1007/s11060-023-04319-1 (PMC10232604; doi:10.1007/s11060-023-04319-1)
Supplement: Supplementary file 3 — Supplementary material 3 (PDF 164.4 kb) [file 11060_2023_4319_MOESM3_ESM.pdf]

Class I HDAC inhibitor entinostat synergizes with PLK1 inhibitors in *MYC*-amplified medulloblastoma cells

Gintvile Valinciute[1,2,15], Jonas Ecker[1-3], Florian Selt[1-3], Thomas Hielscher[5], Romain Sigaud[1,2], Johannes Ridinger[1,2], Venu Thatikonda[1,13,#], Charlotte Gatzweiler[1,2], Sarah Robinson[15], Julie Talbot[6,7], Flavia Bernardi[6,7], Daniel Picard[8,9], Mirjam Blattner-Johnson[1,10], Simone Schmid[11,12], David T. Jones[1,10], Cornelis M. van Tilburg[1-3], David Capper[11,12], Marcel Kool[1,13,14], Marc Remke[8,9], Ina Oehme[1,2], Stefan M. Pfister[1,3,13], Martine F. Roussel[15], Olivier Ayrault[6,7], Olaf Witt[1-3], Till Milde[1-3,*]

1 Hopp Children's Cancer Center Heidelberg (KiTZ), Heidelberg, Germany

2 Clinical Cooperation Unit Pediatric Oncology, German Cancer Research Center (DKFZ) and German Consortium for Translational Research (DKTK), Heidelberg, Germany

3 KiTZ Clinical Trial Unit (ZIPO), Department of Pediatric Hematology and Oncology, Heidelberg University Hospital, Heidelberg, Germany

5 Division of Biostatistics, German Cancer Research Center (DKFZ), Heidelberg, Germany

6 Institut Curie, PSL Research University, CNRS UMR, INSERM, Orsay, France

7 Université Paris Sud, Université Paris-Saclay, CNRS UMR 3347, INSERM U1021, Orsay, France

8 Department of Pediatric Oncology, Hematology and Clinical Immunology, Medical Faculty, University Hospital Düsseldorf, Düsseldorf, Germany

9 Department of Pediatric Neuro-Oncogenomics, German Cancer Research Center (DKFZ), Heidelberg, Germany; and German Cancer Consortium (DKTK), partner site Essen/ Düsseldorf, Düsseldorf, Germany

10 Division of Pediatric Glioma Research, German Cancer Research Center (DKFZ), Heidelberg, Germany

11 Department of Neuropathology, Charité - Universitätsmedizin Berlin, Germany

12 DKTK Partner Site, Berlin, Germany

13 Division of Pediatric Neurooncology, German Cancer Research Center (DKFZ) and German Consortium for Translational Research (DKTK), Heidelberg, Germany

14 Princess Máxima Center for Pediatric Oncology, Utrecht, the Netherlands

15 Department of Tumor Cell Biology, St. Jude Children's Research Hospital, Memphis, TN, USA

# Present address: Global Computational Biology and Digital Sciences, Boehringer Ingelheim RCV GmbH; Co KG, Doktor-Boehringer-Gasse 5-11, 1120 Vienna, Austria

* Corresponding author: Till Milde, Hopp Children's Cancer Center Heidelberg (KiTZ), CCU Pediatric Oncology B310, German Cancer Research Center (DKFZ), Im Neuenheimer Feld 280, 69120 Heidelberg, Germany; Tel.: +49 6221 42 3574; email: t.milde@kitz-heidelberg.de

Supplementary Materials and Methods

Drugs

All inhibitors were stored in accordance with the manufacturers' instructions.

For animal treatment entinostat was purchased as powder (Cat. No. 201266, MedKoo Biosciences Inc., Morrisville, NC, USA) and solved in 2.5 % DMSO (Sigma-Aldrich, Darmstadt, Germany), 1 % Tween-80 (Selleck Chemicals, Houston, TX, USA), 5 % PEG400 (Selleck Chemicals) in saline (B.Braun Medical Inc., Melsungen, Germany). Volasertib was purchased as powder (Cat. No. 200494, MedKoo Biosciences Inc.) and solved in 4 % DMSO, 5 % Tween-80, 5 % PEG400 in saline.

Animal studies

For the survival studies NSG (NOD.Cg-Prkdc^{SCID} Il2rg^{tm1Wjl}/SzJ) mice were purchased from Charles River Laboratory. We used the established PDX model RCMB28 labelled with luciferase. 400 000 cells in 4 µl of NeuroCult^{TM} NS-A Basal medium with human proliferation supplement (Stem Cell Technologies, Vancouver, Canada) were injected into the cerebellum using a 10 µL Hamilton syringe, at the coordinates: bregma -7 mm, lateral 1 mm, depth 2 mm. Tumor growth was monitored by weekly bioluminescence imaging using the IVIS Lumina Series III (PerkinElmer, Waltham, MA, USA). Animals that developed tumors were randomly assigned to 4 groups. Tumor-bearing mice were treated with vehicle (n=4, 5x/week i.p. for entinostat solvent and 3x/week i.v. for volasertib solvent), entinostat (n=8, 5x/week i.p. [10 mg/kg]), volasertib (n=8, 3x/week i.v. [15 mg/kg]) or the combination of entinostat and volasertib (n=8, 5x/week i.p. [10 mg/kg] and 3x/week i.v. [15 mg/kg], respectively). Treatment was started 35 days after implantation once all mice allocated to the 4 groups had shown tumor engraftment. To avoid excessive toxicity usually associated with long exposure to cell cycle kinase inhibitor, treatment was scheduled as follows: 2 weeks treatment, 1 week pause, 2 more weeks treatment. Maximum tolerated doses in combination were established by

monitoring the weight of non-tumor-bearing mice. Mice were euthanized by exposure to CO_2 after the humane endpoint was met.

The *in vivo* on-target effect was determined by treating NSG mice with entinostat (n=4, 5x/week i.p. [10 mg/kg]) or volasertib (n=4, 3x/week i.v. [15 mg/kg]) for 1 week, plus one more dose at day 8 and euthanizing animals after 2 h by exposure to CO_2. Brains were extracted and divided in two: one part was lysed for subsequent immuno-blotting, one part was fixed overnight and embedded in paraffin as described[1]. For volasertib on-target effect determination 100 000 HD-MB03 cells labelled with luciferase[2] were implanted either in the cortices or flanks of 7-week-old female NSG mice obtained from the colony of St. Jude Children's Research Hospital. Mice were treated i.v. either with vehicle or 15 mg/kg volasertib for 1 week (3x/week) and euthanized 4 hours after last injection. Tumors were lysed for immunoblotting.

Short-term PDX cell culture

Group 3 MB cell culture. Patient-derived xenografts (PDXs) from group 3 MB with *MYC* amplification, ICN-MB-PDX-4 and ICN-MB-PDX-7 were generated from primary human MB samples, diagnosed at Children's Necker Hospital in Paris, and transplanted into the cerebellum of immunocompromised NOD/SCID mice[3] and then maintained into the subscapular fat pad of Nude mice. Human samples for xenograft studies were obtained under written informed consent and ethical approved by the Internal Review Board of Necker Sick Children's Hospital, Paris, France.

Tumor cells from group 3 MB models were purified using an enzymatic dissociation followed by a Percoll density gradient separation as previously described[4]. Then, the purified cells were cultured in suspension as spheres, in DMEM/F-12 supplemented with B27, N2, heparin, EGF and bFGF factors (Gibco).

Animal husbandry. Adult immunodeficient NMRI Nude mice and NOD/SCID mice used for development of group 3 MB tumor models were purchased from Janvier Labs. All animals were harbored at the animal facility of the Institut Curie, in accordance with the recommendations of

the European Community (2010/63/UE) for the care and use of laboratory animals. Experimental procedures were specifically approved by the ethics committee of the Institut Curie CEEA-IC #118 (approval number: 03130.02, C91471108 and Authorization APAFiS# 26879-2020081315161665-v1 given by National Authority) in compliance with the international guidelines. And the protocol complied with the internationally established 3R principles, in accordance with the UKCCCR guidelines.

Cell viability assay. For the CellTiter-Glo® Luminescent Cell Viability Assay, Group 3 MB tumor cells were cultured in neurospheres in round bottom 96-well plates and treated with a range of concentrations of either 1) Entinostat or 2) Volasertib, or 3) the combination of both drugs at indicated concentrations. Then, cell viability was evaluated 72 h later using the CellTiter-Glo® Luminescent Cell Viability Assay according to the manufacturer's instructions (Promega Corporation). The results were represented using Bliss independence model to calculate combination indices (CI).

Datasets used for gene expression, ChIP and protein abundance analysis

The gene expression profiles of HD-MB03 cells upon 6 h of 5 µM entinostat treatment used here were generated in our previous study[5]. The genes were ranked according to their statistical significance of differential expression (adjusted p-value and fold change), targetability and advances in clinical development. The ChIP sequencing data for MYC, H3K27ac, and RNA PolII position on chromatin was generated in our previous study and analysed as described[5]. *PLK1* mRNA expression was analysed in the INFORM pediatric cancer dataset[6], and in publicly available array-based or RNA seq-based gene expression datasets accessible via R2: Genomics analysis and visualization platform (https://r2.amc.nl). The following datasets were analysed: Gilbertson (n=76)[7], Pfister (n=223)[8], Kool (n=10), Cavalli (n=763)[9]. The following probes were investigated: 202240_at (PLK1) and 202431_s_at (MYC). PLK1 protein abundance in MB was evaluated in the publicly available Archer[10] and Forget[11] proteomic datasets. *PLK1* mRNA expression effect on patient survival was analysed in publicly available dataset published by Cavalli, et al (n=763)[9]. *PLK1*

very high expression was considered above Q3, *PLK1* very low expression was considered below Q1. Intermediate (between Q1 and Q3) expression was also plotted.

Gene expression profiling and gene set enrichment analysis

Affymetrix CEL files were GCRMA normalized[12] and log2-transformed. Differentially expressed probesets due to treatment were identified using the empirical Bayes approach[13] based on moderated t-statistics as implemented in the Bioconductor package limma[14] accounting for paired samples. Probesets showing an over-additive effect for treatment combination were identified by testing the interaction effect between treatments. Gene set enrichment analysis was performed using the camera test[15]. In case a gene was represented by multiple probesets, the probeset with the strongest effect was selected for GSEA. p-values were adjusted for multiple testing using the Benjamini-Hochberg correction to control the false discovery rate. All analyses were performed with statistical software R 3.6[16].

Statistical analysis and data visualization

Statistical evaluation and data visualization was performed with RStudio v1.4.1717 (RStudio Inc., Boston, MA, USA), GraphPad Prism v5.01 (GraphPad Software, San Diego, CA, USA) and Inkscape 0.92.4 (open source) software. The student's t-test or one-way ANOVA were used as appropriate. A five-parameter logistic model was used for graphically describing the dose-response relationship. IC50 concentrations were calculated using GraphPad Prism v5.01. Combination indices (CI) were calculated using CompuSyn software (ComboSyn Inc., Paramus, NJ, USA) and the Bliss Independence model[17]. Linear regression models and Pearson correlation coefficients were used to evaluate *MYC* and *PLK1* gene expression correlations. The Log Rank test with Bonferroni-Holm multiple testing corrections was used to evaluate the differences between survival curves. p-values <0.05 were considered significant, unless stated otherwise, values were compared to solvent group.

1. Milde T, Lodrini M, Savelyeva L, Korshunov A, Kool M, Brueckner LM, Antunes AS, Oehme I, Pekrun A, Pfister SM, Kulozik AE, Witt O, Deubzer HE (2012) HD-MB03 is a novel Group 3 medulloblastoma model demonstrating sensitivity to histone deacetylase inhibitor treatment. J Neurooncol 110: 335-348 doi:10.1007/s11060-012-0978-1
2. Smith SMC, Bianski BM, Orr BA, Harknett G, Onar-Thomas A, Gilbertson RJ, Merchant TE, Roussel MF, Tinkle CL (2019) Preclinical Modeling of Image-Guided Craniospinal Irradiation for Very-High-Risk Medulloblastoma. Int J Radiat Oncol Biol Phys 103: 728-737 doi:10.1016/j.ijrobp.2018.10.015
3. Morfouace M, Shelat A, Jacus M, Freeman BB, 3rd, Turner D, Robinson S, Zindy F, Wang YD, Finkelstein D, Ayrault O, Bihannic L, Puget S, Li XN, Olson JM, Robinson GW, Guy RK, Stewart CF, Gajjar A, Roussel MF (2014) Pemetrexed and gemcitabine as combination therapy for the treatment of Group3 medulloblastoma. Cancer Cell 25: 516-529 doi:10.1016/j.ccr.2014.02.009
4. Zindy F, Uziel T, Ayrault O, Calabrese C, Valentine M, Rehg JE, Gilbertson RJ, Sherr CJ, Roussel MF (2007) Genetic alterations in mouse medulloblastomas and generation of tumors de novo from primary cerebellar granule neuron precursors. Cancer Res 67: 2676-2684 doi:10.1158/0008-5472.Can-06-3418
5. Ecker J, Thatikonda V, Sigismondo G, Selt F, Valinciute G, Oehme I, Muller C, Buhl JL, Ridinger J, Usta D, Qin N, van Tilburg CM, Herold-Mende C, Remke M, Sahm F, Westermann F, Kool M, Wechsler-Reya RJ, Chavez L, Krijgsveld J, Jager N, Pfister SM, Witt O, Milde T (2021) Reduced chromatin binding of MYC is a key effect of HDAC inhibition in MYC amplified medulloblastoma. Neuro Oncol 23: 226-239 doi:10.1093/neuonc/noaa191
6. Worst BC, van Tilburg CM, Balasubramanian GP, Fiesel P, Witt R, Freitag A, Boudalil M, Previti C, Wolf S, Schmidt S, Chotewutmontri S, Bewerunge-Hudler M, Schick M, Schlesner M, Hutter B, Taylor L, Borst T, Sutter C, Bartram CR, Milde T, Pfaff E, Kulozik AE, von Stackelberg A, Meisel R, Borkhardt A, Reinhardt D, Klusmann JH, Fleischhack G, Tippelt S, Dirksen U, Jurgens H, Kramm CM, von Bueren AO, Westermann F, Fischer M, Burkhardt B, Wossmann W, Nathrath M, Bielack SS, Fruhwald MC, Fulda S, Klingebiel T, Koscielniak E, Schwab M, Tremmel R, Driever PH, Schulte JH, Brors B, von Deimling A, Lichter P, Eggert A, Capper D, Pfister SM, Jones DT, Witt O (2016) Next-generation personalised medicine for high-risk paediatric cancer patients - The INFORM pilot study. Eur J Cancer 65: 91-101 doi:10.1016/j.ejca.2016.06.009
7. Robinson G, Parker M, Kranenburg TA, Lu C, Chen X, Ding L, Phoenix TN, Hedlund E, Wei L, Zhu X, Chalhoub N, Baker SJ, Huether R, Kriwacki R, Curley N, Thiruvenkatam R, Wang J, Wu G, Rusch M, Hong X, Becksfort J, Gupta P, Ma J, Easton J, Vadodaria B, Onar-Thomas A, Lin T, Li S, Pounds S, Paugh S, Zhao D, Kawauchi D, Roussel MF, Finkelstein D, Ellison DW, Lau CC, Bouffet E, Hassall T, Gururangan S, Cohn R, Fulton RS, Fulton LL, Dooling DJ, Ochoa K, Gajjar A, Mardis ER, Wilson RK, Downing JR, Zhang J, Gilbertson RJ (2012) Novel mutations target distinct subgroups of medulloblastoma. Nature 488: 43-48 doi:10.1038/nature11213
8. Northcott PA, Buchhalter I, Morrissy AS, Hovestadt V, Weischenfeldt J, Ehrenberger T, Grobner S, Segura-Wang M, Zichner T, Rudneva VA, Warnatz HJ, Sidiropoulos N, Phillips AH, Schumacher S, Kleinheinz K, Waszak SM, Erkek S, Jones DTW, Worst BC, Kool M, Zapatka M, Jager N, Chavez L, Hutter B, Bieg M, Paramasivam N, Heinold M, Gu Z, Ishaque N, Jager-Schmidt C, Imbusch CD, Jugold A, Hubschmann D, Risch T, Amstislavskiy V, Gonzalez FGR, Weber UD, Wolf S, Robinson GW, Zhou X, Wu G, Finkelstein D, Liu Y, Cavalli FMG, Luu B, Ramaswamy V, Wu X, Koster J, Ryzhova M, Cho YJ, Pomeroy SL, Herold-Mende C, Schuhmann M, Ebinger M, Liau LM, Mora J, McLendon RE, Jabado N, Kumabe T, Chuah E, Ma Y, Moore RA, Mungall AJ, Mungall KL, Thiessen N, Tse K, Wong T, Jones SJM, Witt O, Milde T, Von Deimling A, Capper D, Korshunov A, Yaspo ML, Kriwacki R, Gajjar A, Zhang J, Beroukhim R, Fraenkel E, Korbel JO, Brors B, Schlesner M, Eils R, Marra MA, Pfister SM, Taylor MD, Lichter P (2017) The whole-genome landscape of medulloblastoma subtypes. Nature 547: 311-317 doi:10.1038/nature22973
9. Cavalli FMG, Remke M, Rampasek L, Peacock J, Shih DJH, Luu B, Garzia L, Torchia J, Nor C, Morrissy AS, Agnihotri S, Thompson YY, Kuzan-Fischer CM, Farooq H, Isaev K, Daniels C, Cho BK, Kim SK, Wang KC, Lee JY, Grajkowska WA, Perek-Polnik M, Vasiljevic A, Faure-Conter C, Jouvet A, Giannini

C, Nageswara Rao AA, Li KKW, Ng HK, Eberhart CG, Pollack IF, Hamilton RL, Gillespie GY, Olson JM, Leary S, Weiss WA, Lach B, Chambless LB, Thompson RC, Cooper MK, Vibhakar R, Hauser P, van Veelen MC, Kros JM, French PJ, Ra YS, Kumabe T, Lopez-Aguilar E, Zitterbart K, Sterba J, Finocchiaro G, Massimino M, Van Meir EG, Osuka S, Shofuda T, Klekner A, Zollo M, Leonard JR, Rubin JB, Jabado N, Albrecht S, Mora J, Van Meter TE, Jung S, Moore AS, Hallahan AR, Chan JA, Tirapelli DPC, Carlotti CG, Fouladi M, Pimentel J, Faria CC, Saad AG, Massimi L, Liau LM, Wheeler H, Nakamura H, Elbabaa SK, Perezpena-Diazconti M, Chico Ponce de Leon F, Robinson S, Zapotocky M, Lassaletta A, Huang A, Hawkins CE, Tabori U, Bouffet E, Bartels U, Dirks PB, Rutka JT, Bader GD, Reimand J, Goldenberg A, Ramaswamy V, Taylor MD (2017) Intertumoral Heterogeneity within Medulloblastoma Subgroups. Cancer Cell 31: 737-754 e736 doi:10.1016/j.ccell.2017.05.005

10. Archer TC, Ehrenberger T, Mundt F, Gold MP, Krug K, Mah CK, Mahoney EL, Daniel CJ, LeNail A, Ramamoorthy D, Mertins P, Mani DR, Zhang H, Gillette MA, Clauser K, Noble M, Tang LC, Pierre-François J, Silterra J, Jensen J, Tamayo P, Korshunov A, Pfister SM, Kool M, Northcott PA, Sears RC, Lipton JO, Carr SA, Mesirov JP, Pomeroy SL, Fraenkel E (2018) Proteomics, Post-translational Modifications, and Integrative Analyses Reveal Molecular Heterogeneity within Medulloblastoma Subgroups. Cancer Cell 34: 396-410.e398 doi:10.1016/j.ccell.2018.08.004

11. Forget A, Martignetti L, Puget S, Calzone L, Brabetz S, Picard D, Montagud A, Liva S, Sta A, Dingli F, Arras G, Rivera J, Loew D, Besnard A, Lacombe J, Pages M, Varlet P, Dufour C, Yu H, Mercier AL, Indersie E, Chivet A, Leboucher S, Sieber L, Beccaria K, Gombert M, Meyer FD, Qin N, Bartl J, Chavez L, Okonechnikov K, Sharma T, Thatikonda V, Bourdeaut F, Pouponnot C, Ramaswamy V, Korshunov A, Borkhardt A, Reifenberger G, Poullet P, Taylor MD, Kool M, Pfister SM, Kawauchi D, Barillot E, Remke M, Ayrault O (2018) Aberrant ERBB4-SRC Signaling as a Hallmark of Group 4 Medulloblastoma Revealed by Integrative Phosphoproteomic Profiling. Cancer Cell 34: 379-395 e377 doi:10.1016/j.ccell.2018.08.002

12. Wu Z, Irizarry RA, Gentleman R, Martinez-Murillo F, Spencer F (2004) A Model-Based Background Adjustment for Oligonucleotide Expression Arrays. Journal of the American Statistical Association 99: 909-917

13. Smyth GK (2004) Linear models and empirical bayes methods for assessing differential expression in microarray experiments. Stat Appl Genet Mol Biol 3: Article3 doi:10.2202/1544-6115.1027

14. Smyth GK (2005) limma: Linear Models for Microarray Data. In: Gentleman R, Carey VJ, Huber W, Irizarry RA, Dudoit S (eds) Bioinformatics and Computational Biology Solutions Using R and Bioconductor. Springer New York, New York, NY, pp 397-420

15. Wu D, Smyth GK (2012) Camera: a competitive gene set test accounting for inter-gene correlation. Nucleic Acids Res 40: e133 doi:10.1093/nar/gks461

16. R Core Team (2019) R: A language and environment for statistical computing. R Foundation for Statistical Computing, Vienna, Austria

17. Foucquier J, Guedj M (2015) Analysis of drug combinations: current methodological landscape. Pharmacol Res Perspect 3: e00149 doi:10.1002/prp2.149
